# Comparison of chemical-use between hydraulic fracturing, acidizing, and routine oil and gas development

**DOI:** 10.1371/journal.pone.0175344

**Published:** 2017-04-19

**Authors:** William T. Stringfellow, Mary Kay Camarillo, Jeremy K. Domen, Seth B. C. Shonkoff

**Affiliations:** 1 Earth & Environmental Sciences Area, Lawrence Berkeley National Lab, Berkeley, CA, United States of America; 2 Ecological Engineering Research Program, School of Engineering & Computer Science, University of the Pacific, 3601 Pacific Avenue, Stockton, CA, United States of America; 3 PSE Healthy Energy, 1440 Broadway, Suite 205, Oakland CA, United States of America; 4 Department of Environmental Science, Policy and Management, University of California, Berkeley, Berkeley, CA, United States of America; The University of Akron, UNITED STATES

## Abstract

The potential hazards and risks associated with well-stimulation in unconventional oil and gas development (hydraulic fracturing, acid fracturing, and matrix acidizing) have been investigated and evaluated and federal and state regulations requiring chemical disclosure for well-stimulation have been implemented as part of an overall risk management strategy for unconventional oil and gas development. Similar evaluations for chemicals used in other routine oil and gas development activities, such as maintenance acidizing, gravel packing, and well drilling, have not been previously conducted, in part due to a lack of reliable information concerning on-field chemical-use. In this study, we compare chemical-use between routine activities and the more closely regulated well-stimulation activities using data collected by the South Coast Air Quality Monitoring District (SCAQMD), which mandates the reporting of both unconventional and routine on-field chemical-use for parts of Southern California. Analysis of this data shows that there is significant overlap in chemical-use between so-called unconventional activities and routine activities conducted for well maintenance, well-completion, or rework. A comparison within the SCAQMD shows a significant overlap between both types and amounts of chemicals used for well-stimulation treatments included under State mandatory-disclosure regulations and routine treatments that are not included under State regulations. A comparison between SCAQMD chemical-use for routine treatments and state-wide chemical-use for hydraulic fracturing also showed close similarity in chemical-use between activities covered under chemical disclosure requirements (e.g. hydraulic fracturing) and many other oil and gas field activities. The results of this study indicate regulations and risk assessments focused exclusively on chemicals used in well-stimulation activities may underestimate potential hazard or risk from overall oil field chemical-use.

## Introduction

Scientific, regulatory, and public debates on the environmental and public health dimensions of oil and gas development have been focused on hazardous chemicals used for hydraulic fracturing and other well-stimulation treatments, such as matrix acidizing, that are classified as “unconventional” oil and gas development methods [1–4]. Consequently, new regulations that govern oil and gas development require disclosure of chemical-use during well stimulation activities, but do not require disclosure of chemicals used for any other oil and gas field activities [1,2,4]. However, potentially hazardous chemicals are used throughout the entire oil and gas development process, not just during well stimulations [5–9], so there is interest in examining overall chemical-use on oil and gas fields and comparing chemical-use between regulated “unconventional” development activities and other oil and gas field activities.

Disclosure of chemical-use during well stimulation is considered an important requirement for the protection of human and environmental health, since knowledge of the types and amounts of chemicals used is fundamental to risk assessment [10]. Recent Federal and State regulations mandate chemical disclosures for well stimulations, including hydraulic fracturing and in some cases matrix acidizing and acid fracturing, but reporting chemical-use for other oil and gas field activities, such as well drilling, well completion, well maintenance, and well re-work is not required, unless pressures above the fracture gradient are used [1,2,4,11]. Given the public and scientific concern regarding the use and release of hazardous chemicals during the current oil and gas development boom [12] and the reuse of oil and gas field produced water for beneficial purposes in arid regions [13–15], it is important to evaluate the potential environmental and public health impacts of all chemical additives used in oil and gas development.

Chemicals are used routinely in oil and gas development as part of drilling and cementing of the well casing, repair of formation damage, wellbore clean-outs, scale and corrosion control, and for other production activities. Chemical additives are also used in enhanced oil recovery (EOR) to change fluid properties of oil (e.g. viscosity) and to otherwise increase production of oil within the formation [16]. During well construction, hazardous chemicals may be added to drilling fluids, drilling muds, and cements and are also used to remove debris from wellbores prior to cementing of the annular space between the steel casing and geological formations [9,17]. Chemical additives, including strong acids, are also used for well completion and rework to facilitate hydrocarbon production.

While large numbers and masses of chemical additives are used in routine oil and gas development activities, only a few surveys of routine chemical-use by the oil and gas industry have been conducted [5–8,18]. There is widespread use of potential chemicals of concern, including biocides, quaternary ammonium compounds, and corrosion inhibitors both off-shore and on-shore [5–8,18]. In contrast, several studies examined chemical-use during well stimulation activities, including hydraulic fracturing and matrix acidizing [19–24]. It has been established that chemicals used during well stimulation treatments have environmental pathways of exposure which include accidental spills, reuse of treated produced water, improper zonal isolation of fluids in the subsurface infrastructure and geologies, and discharge of wastewaters to aquatic ecosystems [3,21,24]. It is also known that produced water has similar exposure pathways, so it is of interest to determine overall oil and gas field chemical-use when evaluating the potential environmental and health impacts of oil and gas development.

The reuse of produced water for agricultural purposes is permissible in the western US and produced water is being reused for irrigation, watering livestock, aquifer recharge, and other purposes [13–15,24–26]. In California, produced water from oil fields is used for food crop irrigation, livestock watering, groundwater recharge, and for wetlands and other environmental purposes [15,27]. There are concerns that oil field chemicals or their degradation products will occur in produced water and that these chemicals may pose an unrecognized hazard or risk for produced water beneficial reuse, since potential exposure pathways from beneficial reuse include chemical uptake or deposition on food crops, contamination of regional aquifers through recharge, and the direct contact of farmworkers with produced water [15]. The hazard posed by oil and gas field chemicals would be in addition to other hazards associated with naturally occurring constituents of produced water, such as salts, metals, aromatic hydrocarbons, and naturally occurring radioactive material. The increased interest in reusing produced water [13,28] suggests that the hazards associated with oil and gas field chemicals should be evaluated.

The objective of this study is to assess chemical-use during routine oil and gas development and to compare chemical-use in routine production activities with chemical-use during well stimulation. To our knowledge, only one regulatory agency in the US, the South Coast Air Quality Management District (SCAQMD) in Southern California, requires mandatory disclosure of on-field chemical-use for well drilling, well completion, and well rework activities. These data were used by Abdullah et al. [19] to characterize chemical-use in acidizing. We use these data to compare chemical additive use between well-stimulation (hydraulic fracturing and matrix acidizing treatments) and routine oil field activities to determine similarities and differences in chemical-use. We summarize the chemicals used with respect to frequency of use, masses applied, and toxicity data. Similar data driven approaches have been used previously to evaluate hazards associated with hydraulic fracturing and matrix acidizing [19,21]. The results of our analysis are interpreted in the context of public and scientific concerns about hydraulic fracturing and the beneficial reuse of produced water.

## Methods

Chemical-use data reported to the South Coast Air Quality Management District (SCAQMD) in southern California was analyzed in this study [29]. Under SCAQMD Rule 1148.2, which went into effect on June 4, 2013, operators and chemical suppliers are required to submit and make publicly available chemical usage data related to routine oil and gas activities (well drilling, well completion, and well rework) and well stimulation (hydraulic fracturing, matrix acidizing) in the California counties of San Bernardino, Orange, Riverside, and Los Angeles, including the City of Los Angeles [29]. These counties represent the second most productive oil and gas region in the third largest oil producing state in the United States. Chemical-use for enhanced oil recovery (EOR) and activities beyond upstream oil and gas development such as refining, transmission, and storage are not included in the SCAQMD datasets and are not included in this analysis.

Data on chemical type, mass injected, and water volumes used in oil and gas operations were downloaded from the SCAQMD database for the period of June 4, 2013 to September 2, 2015 [29]. The dataset used for this study consists of 51,514 entries from 1,207 oil and gas “events” conducted at 302 unique locations (identified by latitude and longitude). Events were categorized by operators as well drilling, completion, or rework activities. For completion, activities were further categorized as acidizing, gravel packing, hydraulic fracturing, maintenance acidizing, matrix acidizing, or acid fracturing. In order to focus on routine oil and gas activities, we separated well stimulation events (hydraulic fracturing, matrix acidizing and acid fracturing) from other routine events in our dataset. Entries were edited to standardize chemical names and to validate the assigned Chemical Abstracts Services Registry Number (CASRN). Changes to names of proprietary chemicals that could not be identified by CASRN were limited to correcting obvious spelling errors (e.g., aicd to acid, kerosine to kerosene), changing capitalization, and altering punctuation (e.g. removing dashes). Proprietary chemicals with singular and plural names that indicate chemical mixtures (e.g., ionic surfactant vs ionic surfactants) were maintained as separate entries. In cases where duplicate event IDs were reported, data were consolidated into one event ID entry. In cases where multiple chemical information documents were reported for the same event ID, data were individually assessed and duplicates, where apparent, were deleted.

For the chemical additives identified by CASRN, toxicological data were collected from online chemical databases [30–41]. Computational models within the U.S. EPA EPI Suite software (e.g., BIOWIN) were used to fill data gaps when experimental data were unavailable. Rat, mouse, and rabbit acute oral toxicity data and rat and mouse inhalation toxicity data were collected to represent and compare mammalian toxicity among the chemical constituents. To assess acute environmental toxicity, data for water flea (*Daphnia magna*), fathead minnow (*Pimephales promelas*), rainbow trout (*Oncorhynchus mykiss*), and green algae were collected. Mammalian median lethal dose (LD50) and median lethal concentration (LC50) were used to assess mammalian hazard. Median effective concentration (EC50) and LC50 data were used to assess aquatic species hazard. Toxicity ratings were assigned using the United Nations Globally Harmonized System (GHS) of Classification and Labelling of Chemicals [42]. In the GHS system, lower numbers indicate higher toxicity, with a designation of “1” indicating the most toxic category. When multiple GHS values were available for a given chemical, the lowest value was used. Chemicals for which the LD50, LC50, or EC50 exceeded the least toxic GHS category were classified as non-toxic.

Chemical were identified for further hazard assessment based on frequency of use, median mass of chemical-use per event, and available toxicity data. Frequency of use was calculated by dividing the number of events that utilized a given chemical by the total routine oil and gas events reported in the SCAQMD database. The median mass of chemical usage per event represents the median mass for all events containing that chemical. Where chemical mixtures were reported, individual chemical masses were calculated by multiplying the total mixture mass by the maximum individual chemical concentration. When multiple entries for a given chemical were reported for a single event, the chemical masses were summed within that event.

We compared the chemical-use in routine oil and gas activities in the SCAQMD dataset to hydraulic fracturing chemicals disclosed in the state of California via the voluntary FracFocus chemical disclosure registry, as summarized by Stringfellow et al. [21]. This dataset contains records of chemical use for 1,623 individual hydraulic fracturing operations conducted in California between January 30, 2011 and May 19, 2014. Stringfellow et al. [21] identified 338 unique additives based on name and CASRN combinations, of which 228 were reported with a CASRN and 110 were identified by chemical or common name only or had proprietary designations. The additives included chemicals, mineral proppants and carriers, and base fluids consisting of water, salt, and brine solutions. There were 326 unique additive names identified in the database [21].

## Results and discussion

### Chemical-use in the SCAQMD

In total, 548 chemical additives were used in the SCAQMD between June 2013 and September 2015, with 525 of these being used for routine oil and gas development activities. The most frequently used chemicals include solvents (e.g. methanol), petroleum products (e.g. distillates), and salts (e.g. sodium chloride) that are employed in formulating commercial blends of production chemicals (S1 Table). Also on the list of frequently used chemicals are carboxylic acids (e.g. citric acid and erythorbic acid) used for scale and iron control, biocides, and corrosion inhibitors. For routine acidizing (e.g., acid cleaning for well-maintenance), hydrochloric acid (HCl) and hydrofluoric acid (HF) were used extensively and in large quantities (mean masses of 1,791 and 161 kg per event, respectively). These quantities are consistent with the analysis by Abdullah et al. [19], who reported mean values of 1,908 kg HCl and 175 kg HF per acidizing event (also exclusive of matrix acidizing). Our values may differ due to the different study periods or deletion of duplicate entries by operators. Other additives used in the highest masses include minerals and other chemicals used for gravel packing (e.g. silica), cementing of well casings (e.g. Portland cement and additives), and sealing wells (e.g. bentonite) (S2 Table).

Table 1 is presented as an analysis of chemical use (numbers of chemicals used and masses) by reported activity. There were only a limited number of well-stimulation events in the SCAQMD during this period and no acid fracturing events were reported. Acidizing, maintenance acidizing, well drilling, and gravel packing accounted for the majority of the 1,207 events in the data set (Table 1). Chemical-use for these types of oil and gas field activities is only subject to mandatory reporting in the SCAQMD region.

**Table 1:** Number of chemicals used and their summed masses per event for oil and gas development (does not include water)^a^.

		Chemicals per event				Mass per event (kg)			
Pooled Activities	Events^a^	Mean	Median	Min	Max	Mean	Median	Min	Max
Acidizing	256	25	20	1	41	4,132	3,459	10	24,043
Gravel packing	169	6	3	1	65	24,655	6,297	61	710,722
Hydraulic fracturing	13	25	23	15	37	129,910	142,245	4,526	243,219
Maintenance acidizing	390	30	35	2	52	2,779	2,028	155	15,548
Maintenance acidizing and gravel packing	3	27	27	27	27	7,712	6,632	6,518	9,985
Matrix acidizing	7	21	20	20	23	4,210	3,055	1,970	10,791
Well completion and rework—type not specified	43	20	21	1	71	16,287	8,028	215	100,566
Well drilling	186	46	54	3	72	1,828,619	97,669	96	309,284,305
Well drilling with gravel packing	136	57	58	26	66	239,305	181,098	21,552	1,233,365

^a^There are 1,207 events in the data set but four events have only water listed so they are not included in this table (N = 1,203).

### Comparison of chemical-use between routine activities and well-stimulation treatments within the SCAQMD

**Fig 1:**
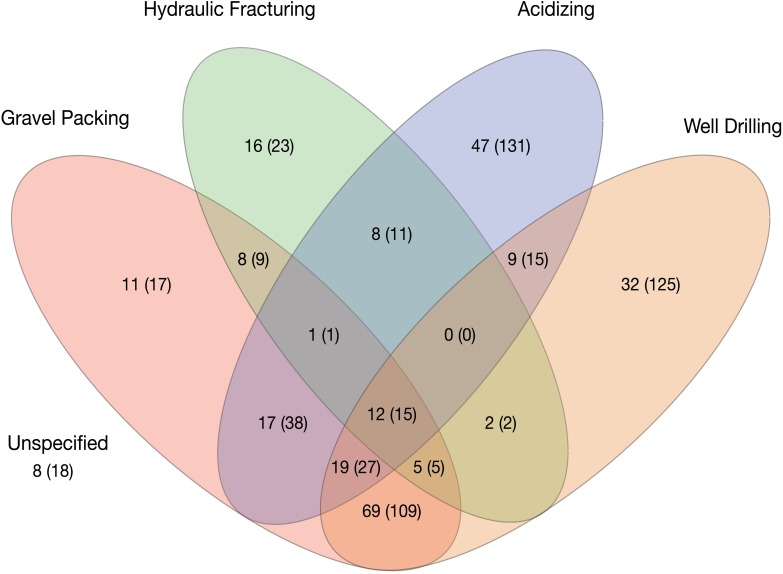
Venn diagram showing number of chemicals used in oil and gas production. The first number represents chemicals with CASRN and the number in parentheses represents the total number of reported chemicals. Does not include base fluids. Acidizing includes matrix acidizing, acidizing, and maintenance acidizing.

Overall, a large number of constituents were used in both routine activities and well-stimulation activities and chemicals were applied in large masses (Table 1). The masses used in hydraulic fracturing were high because of the large quantities of proppants used. Similarly, well drilling uses large quantities of Portland cement and minerals for well construction. Comparison of the chemicals used for different on-field activities showed significant overlap in the chemicals used for hydraulic fracturing and routine oil and gas development operations (Fig 1). Only 23 (4.2%) chemicals were used exclusively for hydraulic fracturing in the SCAQMD. However, the SCAQMD dataset includes only a small number of hydraulic fracturing operations (13) and the degree of overlap in chemical use between different oil field operations may not be representative of other regions. A comparison of chemical use for routine oil and gas development as reported in the SCAQMD database and chemical use for fracturing in the whole state of California, indicates the degree of overlap is less.

**Fig 2:**
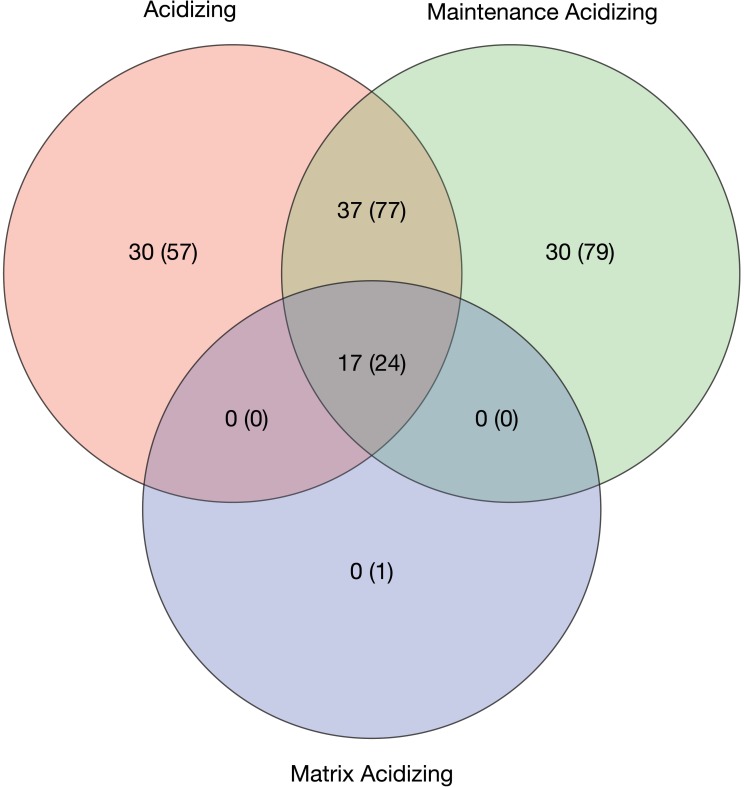
Venn diagram showing number of chemicals used for acidizing operations (routine and well stimulation). The first number represents chemicals with CASRN and the number in parentheses represents the total number of reported chemicals. Does not include base fluids.

Examining different types of acidizing within the SCAQMD, the median numbers of chemicals used in routine acidizing (20 for acidizing and 35 for maintenance acidizing) were similar in number to the median value of 20 used in matrix acidizing (Table 1). An analysis of chemicals used for acid treatments shows that there is considerable overlap in the chemicals used for the different applications of acid (Fig 2). The one compound used exclusively for matrix acidizing was identified only as “DDBSA salt,” presumably a dodecylbenzenesulfonic acid salt, but without a corresponding CASRN, this identification is tentative. Maintenance acidizing used a lower median mass of chemicals (2,028 kg) than treatments reported as acidizing (3,459 kg) or matrix acidizing (3,055 kg). These quantities demonstrate that additives usage in other acidizing is not appreciably different than what is used in matrix acidizing (classified as well stimulation).

**Fig 3:**
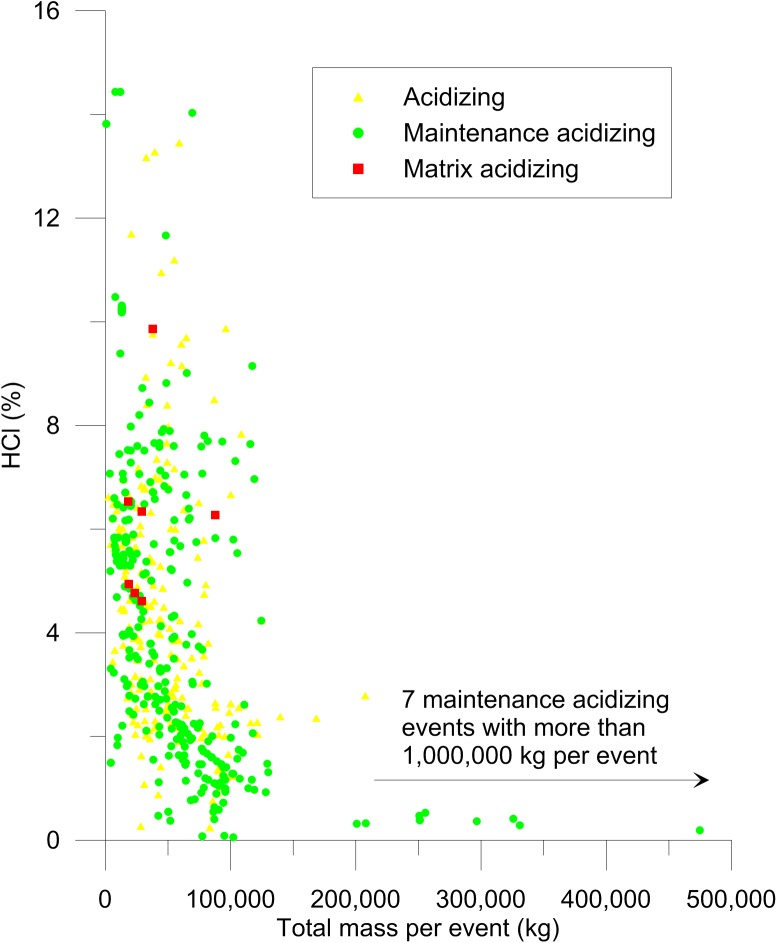
Concentrations of hydrochloric acid (HCl) used in acidizing. Sixteen events where water was not reported were excluded because the concentrations could not be calculated.

**Fig 4:**
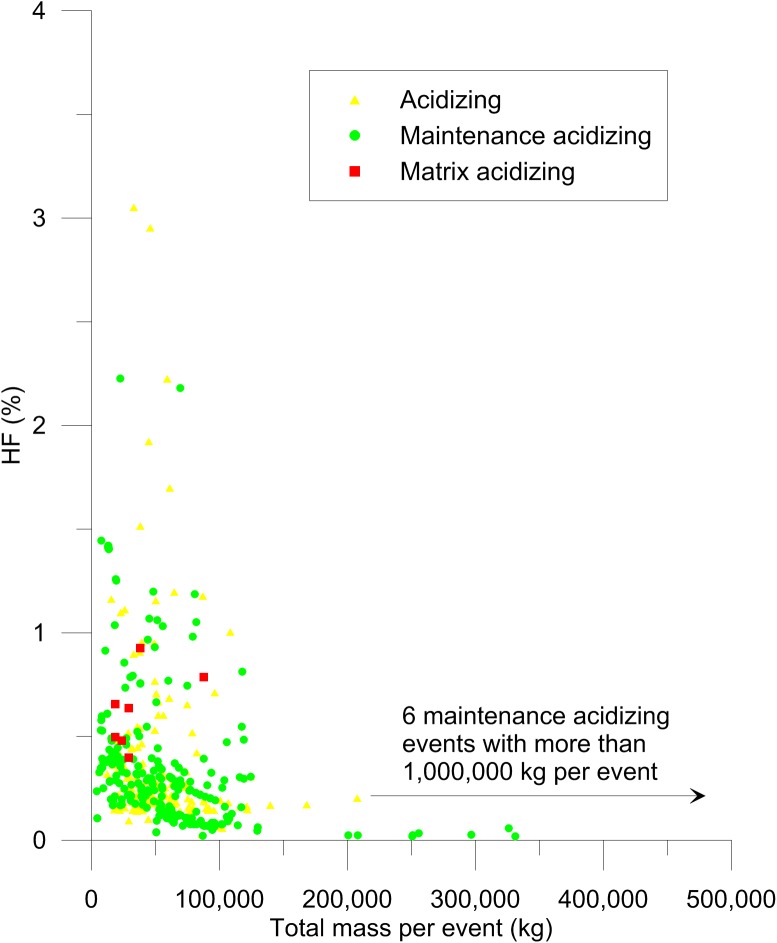
Concentrations of hydrofluoric acid (HF) used in acidizing. Sixteen events where water was not reported were excluded because the concentrations could not be calculated.

Concentrations of hydrochloric acid (HCl) and hydrofluoric acid (HF) used in all types of acidizing events were similar, as were the total masses of additives used (Figs 3 and 4). Hydrochloric acid concentrations ranged from approximately 0–15% (Fig 3) while HF concentrations were approximately 0–3% (Fig 4). In California, the distinction between routine acidizing and acid stimulation (matrix acidizing and acid fracturing) is based on calculation of the acid threshold volume that is determined based on wellbore volume and formation porosity [1]. The acid threshold volume cannot be calculated without site-specific information that is not reported to the publically available SCAQMD database. However, it is apparent that large quantities of acid and high concentrations are being used in all types of acidizing events. Since there is clear overlap in concentrations and amounts of acid used for events reported as matrix acidizing, which are potentially regulated by state law, and routine maintenance acidizing (Figs 3 and 4), these results suggest that regulations focused only on disclosures of chemicals used in well stimulation events may not be sufficiently protective of public or environmental health.

### Comparison of chemical-use between routine oil and gas development activities in the SCAQMD and hydraulic fracturing throughout California

The number of chemicals used in routine oil and gas development activities in the SCAQMD is as high or higher than the number of chemicals used for hydraulic fracturing throughout the State of California [21]. In Stringfellow et al. [21], 338 unique chemical additives were identified as used in hydraulic fracturing fluids in California, with 228 of these identified by CASRN. These data were reported voluntarily by industry, but are believed to be representative of hydraulic fracturing as practiced in California [21,24,43,44]. Here, we identified 525 additives used in routine oil and gas production, with 249 identified by CASRN. In Stringfellow et al. [21], there was a median of 23 components per hydraulic fracturing treatment, inclusive of base fluids and proppants. In the SCAQMD, the number of additives per event varied by activity (Table 1). The median number of chemical additives was as low as three for gravel packing and the median number of chemical additives used in well drilling was much higher (54).

In the SCAQMD, the median mass used per hydraulic fracturing event was high (142,245 kg), but when water and quartz sand proppants were removed, the median mass of chemical additives was 6,725 kg. This is approximately three times higher than the value of 2,057 kg obtained by Stringfellow et al. [21], who analyzed voluntarily reported data from the whole state of California. This difference may be attributed to differences in regional reservoir geology between the SCAQMD and the rest of California [44] and corresponding hydraulic fracturing practices: most of the data analyzed by Stringfellow et al. [21] was reported from Kern County, CA while the data here originated primarily from Orange and Los Angeles Counties.

Of the 249 chemicals identified by CASRN that are used for routine oil and gas development in the SCAQMD (Table 2), 124 (24%) were identified by Stringfellow et al. [21] as being used for hydraulic fracturing in California, further demonstrating overlap in chemical usage between hydraulic fracturing and routine activities. Further examination of the types of chemicals used in routine oil and gas development activities and in hydraulic fracturing yields both similarities and differences. As an example, ten biocides were identified in the hydraulic fracturing data set reported by Stringfellow et al. [21] while only six were identified here as used in routine activities. The biocides were used in 63% of routine activities conducted in the SCAQMD compared to 93% of hydraulic fracturing treatments [21]. In routine use, the most commonly used biocides were formaldehyde, used in 677 (57%) events, and glutaraldehyde, used in 274 (23%) events. In the hydraulic fracturing treatments, isothiazolones were used in 73% of treatments [21,24]. This demonstrates that biocides are used extensively in different types of oil and gas production activities.

**Table 2:** Data availability for chemicals used in routine oil and gas development.

Number of chemicals	Proportion of all chemicals	CASRN	Mass data	Toxicity data
151	30%	Available	Available	Available^a^
1	0%	Available	Unavailable	Available^a^
97	18%	Available	Available	Unavailable^a^
43	8%	Unavailable	Available	Unavailable
233	44%	Unavailable	Unavailable	Unavailable

^a^Does not include EPI Suite computational estimates for green algae ecotoxicity

Corrosion inhibitors were used more extensively in routine operations than in hydraulic fracturing treatments. Ten corrosion inhibitors were identified in both the current data set and in the hydraulic fracturing data set [21], although the numbers are likely higher since many chemicals used as corrosion inhibitors also have other functions in oil and gas production (e.g. surfactants). In routine operations in the SCAQMD, corrosion inhibitors were used in 894 events (75% of all events), but they were only used in 6% of the hydraulic fracturing treatments [21]. The prevalent use of corrosion inhibitors in the SCAQMD is not surprising given the common use of strong acids in well maintenance and completion activities.

The substantial overlap between chemicals used in hydraulic fracturing fluids and those used in routine oil and gas development processes clearly demonstrate that the regulatory focus on reporting chemical-use for well-stimulation activities (e.g. hydraulic fracturing) to the exclusion of routine maintenance activities (e.g. wellbore cleaning) does not fully address potential environmental and public health concerns from on field chemical-use, particularly in the context of beneficial reuse of produced water for agriculture [15]. A more complete understanding of chemical usage–including type; toxicity and environmental profile; and mass, timing, frequency used–in routine oil and gas development is needed to support decision making with respect to beneficial reuse of produced water and this study contributes to filling this data gap.

### Comparison of chemical-use between routine oil and gas development activities in the SCAQMD and other oil and gas fields throughout the U.S. and World

It is difficult to determine with certainty if chemical use on oil fields in the SCAQMD is representative of chemical-use on oil fields throughout the U.S. or the world, since data on chemical-use is rarely collected by governments or published by industry. Hudgins analyzed and published chemical-use data provided voluntarily by off-shore operations in the Gulf of Mexico [7] and the North Sea [8]. Comparison of the Hudgins’ studies with chemicals used in the SCAQMD shows that chemicals are used for common purposes, such as microbial control, scale control, and cleaning, at all locations [7,8]. Hudgins’ studies did not identify chemicals by CASRN, but some chemicals were identified sufficiently by name to allow positive identification of 47 chemicals from the North Sea study [8] and 25 chemicals from the Gulf of Mexico study [7]. Thirty-five chemicals could be positively identified as being used in both the North Sea and in the SCAQMD and 15 were positively identified as being used in both the Gulf of Mexico and the SCAQMD. Overall, these results, combined with a review of industrial literature, patents, and other sources, suggests that many of the chemicals used on the SCAQMD, or closely related compounds, would be found on oil fields worldwide [5–8,19–22,45].

### Analysis of chemical hazards using data science approaches

One of the important requirements of regulations directed at oil and gas development and production is the disclosure of the types and amounts of chemicals used on-field [1,2,4,11,46]. Chemical disclosure is widely recognized as a fundamental prerequisite for the open and transparent analysis of the hazards and risks associated with chemicals [2,4,10,27,45,46]. Previous studies have shown that many oil and gas field chemicals are not expected to have negative environmental or health impacts, but that some compounds, including surfactants, biocides, and corrosion inhibitors may be harmful to the environment, and that in many cases there is insufficient information to confidentially evaluate the potential environmental impact of chemicals that are used in significant amounts on oil and gas fields [19–24,47,48].

A preliminary hazard assessment for oil field chemicals being used in the SCAQMD was conducted using data science methods applied against hydraulic fracturing chemicals [20,21]. As shown in Table 2, 52% of the chemicals used in the SCAQMD were reported without a CASRN and could therefore not be evaluated using a data science approach, which requires CASRN to match compounds with corresponding environmental and toxicity information. Of the 53 chemicals used most frequently (top 10%), 18 were reported without a corresponding CASRN. The top 10% of the chemicals used in the highest median masses per event also did not always have associated CASRN (S2 Table). For example, the fourth most commonly used additive is a proprietary chemical identified only as “polyoxyalkylenes,” which could be any one of potentially hundreds of chemicals or chemical formulations. Compounds reported by CASRN mostly had corresponding mass-usage information, important for risk analysis, but 97 did not have toxicity profiles in the public databases used in this study (Table 2; S3 Table). Altogether, 70% of the chemical additives reported in the SCAQMD could not be fully evaluated using data-based hazard analysis approaches [20,21,47], suggesting that current reporting requirements may need to be strengthened, if the regulatory objective includes generating data needed for risk assessments.

Analysis of chemicals by mammalian toxicity revealed that five chemicals were classified as GHS Category 2 contaminants based on acute mammalian oral exposure and 13 were classified as GHS Category 1 or 2 for acute mammalian inhalation toxicity (Table 3). These results are similar to results found by Stringfellow et al. [21] for hydraulic fracturing operations. Several of the most toxic chemicals identified are biocides: 5-chloro-2-methyl-3(2H)-isothiazolone, DBNPA (2,2-dibromo-3-nitrilopropionamide), formaldehyde, and glutaraldehyde. Corrosion inhibitors are also represented on the list of most toxic chemical additives: propargyl alcohol and thioglycolic acid (Table 3). Mammalian toxicity data were unavailable for 105 (42%) of the 249 chemicals with CASRN.

**Table 3:** Chemicals used in routine oil and gas development that are classified by the United Nations Globally Harmonized System (GHS) Categories 1 and 2 for acute mammalian toxicity^a^.

Chemical name	CASRN	Oral toxicity ratings			Inhalation toxicity ratings		Frequency of use (% events)	Median mass per event (kg)
		Rat	Mouse	Rabbit	Rat	Mouse		
2-Butoxyethanol (Ethylene glycol butyl ether)	111-76-2	4	4	3	2	-	26.5%	545
5-Chloro-2-methyl-3(2H)-isothiazolone	26172-55-4	4	-	-	2	-	0.1%	5.2
DBNPA (2,2-dibromo-3-nitrilopropionamide)	10222-01-2	3	-	3	1	-	0.3%	4.1
Ethylene oxide	75-21-8	3	3	-	2	3	1.0%	<0.1
Ferric chloride	7705-08-0	2	4	-	-	-	0.5%	30
Formaldehyde	50-00-0	2	2	-	2	2	57.0%	<0.1
Glutaraldehyde	111-30-8	3	3	-	1	-	23.1%	75
Glycolic acid	79-14-1	4	4	-	1	-	0.1%	89
Hydrofluoric acid	7664-39-3	-	-	-	2	2	43.6%	96
Lithium hydroxide	1310-65-2	3	4	-	2	-	0.2%	22
Petroleum distillates	64741-44-2	-	-	-	2	-	0.1%	138,679
Propargyl alcohol	107-19-7	2	2	-	3	-	53.8%	3.7
Sulfuric acid	7664-93-9	5	-	-	2	-	2.1%	<0.1
Tetrasodium ethylenediaminetetraacetate	64-02-8	4	2	-	-	-	0.3%	<0.1
Thioglycolic acid	68-11-1	3	3	3	1	-	0.1%	98
Toluene	108-88-3	4	-	-	>4	2	1.4%	6.7
Zinc sulfate	7733-02-0	3	2	4	-	-	0.2%	50

^a^Only chemicals with valid CASRN could be evaluated.

Analysis of ecotoxicity characteristics of the chemicals revealed that 58 chemical additives were classified as GHS Category 1 or 2 (Table 4). Twenty-six of these classifications were determined using computational estimates from the U.S. EPA Ecological Structure Activity Relationships (ECOSAR) software for green algae ecotoxicity, available through EPI Suite. The remainder of the ecotoxicity determinations were made using experimental data. A wide range of chemicals were identified as being toxic to aquatic organisms. The list includes acids, hydrocarbons, biocides, corrosion inhibitors, surfactants, and other industrial chemicals (e.g. tall oil). Experimental ecotoxicity data were unavailable for 146 (59%) of the 249 chemicals with CASRN; when ECOSAR estimates were included, ecotoxicity data were unavailable for 129 (52%) chemicals with CASRN.

**Table 4:** Chemicals used in routine oil and gas development that are classified by the United Nations Globally Harmonized System (GHS) in Categories 1 and 2 for ecotoxicity^a^.

Chemical name	CASRN	Water Flea^b^	Fathead Minnow^c^	Rainbow Trout^d^	Green Algae^e^	Frequency of use (% events)	Median mass per event (kg)
1,2,3-Trimethylbenzene	526-73-8	-	-	-	2	0.3%	1.0
1,2,4-Trimethylbenzene	95-63-6	2	2	-	2	5.7%	1.6
1,3,5-Trimethylbenzene	108-67-8	2	-	-	2	0.3%	2.3
2-Mercaptoethyl alcohol	60-24-2	2	-	-	2	0.7%	2.5
2-Methyl-3(2H)-isothiazolone	2682-20-4	1	-	1	1	0.2%	2.6
5-Chloro-2-methyl-3(2H)-isothiazolone	26172-55-4	1	-	1	1	0.1%	5.2
Acrylamide	79-06-1	3	>3	>3	1	0.8%	<0.1
Alcohols, C10-14, ethoxylated	66455-15-0	-	-	-	1	0.6%	64
Aluminum	7429-90-5	-	-	1	-	16.5%	9.1
Ammonium chloride	12125-02-9	>3	2	>3	-	48.4%	454
Benzene, c10-c16 alkyl derivatives	68648-87-3	-	-	-	1	0.9%	<0.1
Benzene, tetrapropylene-	25265-78-5	-	-	-	1	0.1%	2.7
Benzoisothiazolinone	2634-33-5	1	-	1	1	0.1%	<0.1
Bis(isopropyl)naphthalene	38640-62-9	-	-	-	1	2.0%	1.8
Canola oil	120962-03-0	-	-	-	1	0.3%	92
Cocamidopropyl betaine	61789-40-0	2	-	-	>3	0.7%	<0.1
Cyclohexasiloxane, 2,2,4,4,6,6,8,8,10,10,12,12-dodecamethyl-	540-97-6	-	-	-	1	0.3%	<0.1
Cyclopentasiloxane, 2,2,4,4,6,6,8,8,10,10-decamethyl-	541-02-6	-	-	-	1	0.3%	<0.1
DBNPA (2,2-dibromo-3-nitrilopropionamide)	10222-01-2	1	1	1	1	0.3%	4.1
Dodecylbenzene	123-01-3	-	-	-	1	0.1%	5.4
Dodecylbenzene sulfonic acid	27176-87-0	2	-	2	3	1.4%	<0.1
Ethanesulfonic acid, 2-[methyl[(9z)-1-oxo-9-octadecen-1-yl]amino]-, sodium salt (1:1)	137-20-2	-	-	-	2	0.6%	53
Ethoxylated C14-15 alcohols	68951-67-7	1	1	1	1	1.3%	2.4
Ethoxylated hexanol	68439-45-2	2	-	2	>3	0.3%	16
Ethylbenzene	100-41-4	2	2	2	2	31.3%	2.9
Fatty acids, tall-oil	61790-12-3	>3	-	-	1	0.4%	7.1
Fatty acids, tall-oil, reaction products with triethanolamine	67784-78-5	-	-	-	2	1.3%	<0.1
Ferric chloride	7705-08-0	2	3	-	-	0.5%	30
Glutaraldehyde	111-30-8	1	2	2	2	23.1%	75
Glyoxal	107-22-2	>3	>3	0	2	23.0%	3.6
Hydrochloric acid	7647-01-0	1	-	2	-	54.8%	1,311
Hydrotreated light petroleum distillate	64742-47-8	-	3	2	1	32.9%	17
Isopropylbenzene	98-82-8	3	2	2	2	29.5%	0.3
Lecithins	8002-43-5	-	-	-	1	0.3%	1.4
Lithium hypochlorite	13840-33-0	1	-	1	-	0.2%	129
Naphtha (petroleum), heavy catalytic reformed	64741-68-0	-	-	-	2	0.2%	18
Naphthalene	91-20-3	1	1	1	2	48.4%	0.3
Octamethylcyclotetrasiloxane	556-67-2	-	-	-	1	0.3%	<0.1
Petroleum distillate-mineral oil grade	8002-05-9	1	-	-	1	0.1%	30
Petroleum distillates	64741-44-2	-	-	-	1	0.1%	138,679
Petroleum distillates	64742-46-7	-	-	-	1	0.1%	138,679
Poly(oxy-1,2-ethandiyl), a-(nonylphenyl)-w-hydroxy-	9016-45-9	2	-	2	2	13.2%	4.6
Polyethylene glycol monostearate	9004-99-3	-	-	-	1	1.3%	<0.1
Polypropylene	9003-07-0	-	-	-	1	1.1%	56
Polysiloxanes, di-Me	63148-62-9	3	-	-	1	1.6%	<0.1
Propargyl alcohol	107-19-7	-	2	-	>3	53.8%	3.7
Quinoline	91-22-5	3	1	-	3	18.8%	0.1
Sodium chloroacetate	3926-62-3	>3	-	-	1	0.3%	<0.1
Sodium hypochlorite	7681-52-9	1	1	1	>3	0.2%	2.3
Sodium silicate	1344-09-8	1	-	-	-	0.7%	72
Solvent naphtha, petroleum, heavy arom.	64742-94-5	1	3	2	2	39.0%	1.8
Solvent naphtha, petroleum, light arom.	64742-95-6	2	-	2	2	5.8%	1.7
Sorbitan monostearate	1338-41-6	-	-	-	1	1.3%	<0.1
Stearic acid	57-11-4	-	-	-	1	12.1%	150
Sulfonic acids, c14-16-alkane hydroxy and c14-16-alkene, sodium salts	68439-57-6	2	-	-	3	0.1%	5.4
Tall oil	8002-26-4	-	-	-	1	0.8%	13
Xylenes	1330-20-7	-	3	2	2	32.0%	1.5
Zinc sulfate	7733-02-0	1	1	1	-	0.2%	50

^a^Only chemicals with valid CASRN could be evaluated.

^b^Daphnia magna

^c^Pimephales promelas

^d^Oncorhynchus mykiss

^e^computational estimates from EPI Suite.

Although a complete risk assessment is beyond the scope of this study, evaluation of the frequency of chemical use and the mass of chemical used can provide context for the potential risk associated with the use of hazardous chemicals. Of the 17 chemicals with high mammalian toxicity only four of these were used in more than 25% of events (Table 3). Quantities of the most toxic chemicals used varied. Seven of the toxic chemicals were used in median quantities of less than 10 kg per treatment, while nine were used in larger amounts. Glutaraldehyde (used in 23% of events) was applied with a median quantity per treatment of 75 kg. While formaldehyde was used more frequently (57% of events), the median quantity added was less than 1 kg per treatment. The complexity of toxicity information, paired with data on frequency of use and quantities applied (Table 3), suggest that while hazard assessments such as this as useful for characterizing chemical-use, more detailed risk assessments are needed.

Nine of the most toxic chemicals from an aquatic perspective were used in more than 25% of events (Table 4). The most frequently used chemicals on the list were hydrochloric acid, propargyl alcohol, ammonium chloride, and naphthalene, used in 48% of events or more. Propargyl alcohol and naphthalene were used in small quantities (median masses of less than 5 kg per treatment) although hydrochloric acid and ammonium chloride were used in much higher amounts (median masses of 1,311 and 454 kg per treatment). The higher number of chemical additives posing ecotoxicity issues and the frequent use of these chemicals, suggests that the ecosystem risks need to be fully evaluated in produced water reuse projects.

### Evaluation of chemical hazards using regulatory lists

To further investigate the potential hazards associated with chemicals used in routine oil and gas development activities, six regulatory lists were referenced (S4 Table). The result of the comparison with these regulatory lists was that twenty-two of the chemicals were on the California Toxic Air Contaminant List [41], 12 were on the California Proposition 65 List [40], 10 were on the U.S. EPA Drinking Water Standards and Health Advisories List [49], six were present on the U.S. EPA Contaminant Candidate List 4 [50], three were on the European Chemicals Agency Substance of Very High Concern Candidate List [51], and two were on the OSPAR List of Substances of Possible Concern [45]. These results demonstrate that some of the chemicals used in routine oil and gas development activities are chemicals of concern, as identified by multiple state, federal, and international environmental agencies due to their toxicities. However, the actual risk proposed by these chemicals would need to be determined in the context of their use and potential release into the environment.

It should be noted that comparison with regulatory lists also indicate that many of the chemicals used in the SCAQMD are expected to present little or no human health or ecotoxicity hazard, even if discharged into the environment. Of the chemicals reported with CASRN, 56 are on the OSPAR list of chemicals not expected to pose environmental harm [22]. These chemicals include inert minerals (e.g. silica, graphite, mica, diatomaceous earth), common salts (e.g. calcium carbonate, calcium chloride, sodium carbonate, etc.), chemicals that rapidly degrade in the environment (e.g. acetic acid, ethylene glycol, 1-butanol), and food additives (e.g. xanthan gum, guar gum, sodium erythorbate, starch).

## Conclusions

In this study we compared routine oil and gas field chemical use, which is not typically subject to disclosure regulations, with chemical use for hydraulic fracturing and other well stimulation techniques that are subject to regulation mandating chemical disclosure. Our results indicate that there is substantial overlap between the chemicals used in well stimulation and those used in routine oil and gas development activities. Similarities were observed in the numbers of chemicals used, the masses in which they were applied, the frequency of use, and their toxicological profiles. Our analysis shows that hydraulic fracturing is just one of many applications of hazardous chemicals on oil and gas fields and suggests that limiting disclosure requirements for oil and gas field chemical-use to hydraulic fracturing and other well-stimulation events may not be fully protective of human and environmental health, especially in the context of beneficial reuse of produced water for irrigation, wildlife, livestock watering, and groundwater recharge.

## Supporting information

S1 Table Constituents used for routine oil and gas development activities (exclusive of well stimulation) in the SCAQMD, June 4, 2013 to September 2, 2015, sorted by frequency of use. Total number of events is 1,187.
(PDF)

S2 Table The top 10% median masses of additives used in routine oil and gas development activities (exclusive of well stimulation) in the SCAQMD, June 4, 2013 to September 2, 2015. Total number of events is 1,187.
(PDF)

S3 Table Chemicals reported to the SCAQMD and used in routine oil and gas production for which experimental toxicity information could not be located (N = 97). (PDF)

S4 Table Chemicals reported to the SCAQMD and used in routine oil and gas development activities considered chemicals of concern based on six reference lists consulted. (PDF)
