# Supplementary material for: Comparison of chemical-use between hydraulic fracturing, acidizing, and routine oil and gas development
Source: PLoS One. 2017 Apr 19;12(4):e0175344. doi: 10.1371/journal.pone.0175344 (PMC5396893; doi:10.1371/journal.pone.0175344)
Supplement: S1 Table — Total number of events is 1,187. (PDF) [file pone.0175344.s001.pdf]

S1 Table. Constituents used for routine oil and gas development activities (exclusive of well stimulation) in the SCAQMD, June 4, 2013 to September 2, 2015, sorted by frequency of use. Total number of events is 1,187.

Rank	Constituent	CASRN	Freq. of use (%)	Total mass (kg)	Median mass per event (kg)	Mean mass per event (kg)	Min mass per event (kg)	Max mass per event (kg)
1	Water base fluid	7732-18-5	97.6%	196,128,245	48,118	169,515	<0.1	4,483,250
2	Methanol	67-56-1	75.7%	43,898	20	49	<0.1	811
3	Sodium chloride	7647-14-5	74.9%	1,647,927	10	1,854	<0.1	453,835
4	Polyoxyalkylenes	Proprietary	65.4%	--	--	--	--	--
5	Citric acid	77-92-9	60.1%	98,553	91	133	0.1	7,034
6	Formaldehyde	50-00-0	57.0%	33	<0.1	<0.1	<0.1	1.9
7	Hydrochloric acid	7647-01-0	54.8%	1,165,757	1,311	1,791	10	12,052
8	Propargyl alcohol	107-19-7	53.8%	2,926	3.7	4.6	<0.1	24
9	Ammonium chloride	12125-02-9	48.4%	421,116	454	732	<0.1	18,847
10	Naphthalene	91-20-3	48.4%	503	0.3	0.9	<0.1	281
11	Hydrofluoric acid	7664-39-3	43.6%	83,007	96	161	0.3	1,513
12	Olefin	Proprietary	43.1%	--	--	--	--	--
13	Erythorbic acid	89-65-6	42.9%	8,684	13	17	0.9	110
14	Fatty acids	Proprietary	42.9%	--	--	--	--	--
15	Citrus terpenes	94266-47-4	41.6%	43,907	57	89	4.8	814
16	Hydrocarbons, terpene processing by-products	68956-56-9	40.7%	29,524	40	61	3.4	581
17	Potassium chloride	7447-40-7	40.2%	28,907,629	7,035	60,603	<0.1	1,136,781
18	Crystalline silica quartz	14808-60-7	40.1%	3,917,132	7,165	8,229	6.5	65,385
19	Thiourea, polymer with formaldehyde and 1-phenylethanone	68527-49-1	39.3%	6,154	10	13	0.7	52
20	Solvent naphtha, petroleum, heavy arom.	64742-94-5	39.0%	12,332	1.8	27	0.2	2,807
21	Hydrotreated light petroleum distillate	64742-47-8	32.9%	74,469	17	191	0.9	38,400
22	Xylenes	1330-20-7	32.0%	80,029	1.5	211	<0.1	11,510
23	Ethylbenzene	100-41-4	31.3%	14,971	2.9	40	0.4	2,579
24	Aromatic hydrocarbon (1)	Proprietary	30.6%	--	--	--	--	--
25	Aromatic hydrocarbon (2)	Proprietary	30.6%	--	--	--	--	--
26	Oxyalkylated alkylphenol	Proprietary	30.2%	--	--	--	--	--

Rank	Constituent	CASRN	Freq. of use (%)	Total mass (kg)	Median mass per event (kg)	Mean mass per event (kg)	Min mass per event (kg)	Max mass per event (kg)
27	Oxyalkylated amine quat	Proprietary	30.2%	--	--	--	--	--
28	Isopropylbenzene	98-82-8	29.5%	126	0.3	0.4	<0.1	5.7
29	Oxyalkylated alkylphenolic resin	Proprietary	27.9%	--	--	--	--	--
30	Inorganic compound	Proprietary	27.8%	--	--	--	--	--
31	Crosslinked polyol ester	Proprietary	27.6%	--	--	--	--	--
32	Polyglycol ester	Proprietary	27.6%	--	--	--	--	--
33	Aromatic hydrocarbon (3A)	Proprietary	27.5%	--	--	--	--	--
34	Aromatic hydrocarbon (3B)	Proprietary	27.5%	--	--	--	--	--
35	Aromatic hydrocarbon (3C)	Proprietary	27.5%	--	--	--	--	--
36	Diol compound	Proprietary	27.5%	--	--	--	--	--
37	2-Butoxyethanol (ethylene glycol butyl ether)	111-76-2	26.5%	46,834	55	149	<0.1	3,453
38	Xanthan gum	11138-66-2	24.9%	123,975	346	419	<0.1	3,059
39	Portland cement	65997-15-1	24.9%	7,427,597	22,481	25,178	93	106,584
40	Bentonite	1302-78-9	24.8%	708,757	570	2,427	7.3	217,271
41	Calcium oxide	1305-78-8	24.4%	440,651	1,433	1,520	5.2	7,413
42	Gypsum	13397-24-5	24.3%	310,276,445	2,771	1,073,621	9.1	309,126,252
43	Magnesium oxide	1309-48-4	24.3%	445,681	1,474	1,542	2.1	7,230
44	Barium sulfate	7727-43-7	23.9%	7,639,223	20,544	26,899	14	540,586
45	Disodium metasilicate	6834-92-0	23.4%	93,416	367	336	0.4	10,135
46	Sulfonate	Proprietary	23.2%	500,337	1,746	1,939	68	10,047
47	Glutaraldehyde	111-30-8	23.1%	39,856	75	146	<0.1	1,374
48	Sodium bicarbonate	144-55-8	23.1%	121,572	340	449	1.5	2,245
49	Glyoxal	107-22-2	23.0%	2,309	3.6	8.5	<0.1	1,178
50	Cellulose, carboxymethyl ether, sodium salt	9004-32-4	22.7%	287,308	953	1,068	23	6,622
51	Acetic acid ethenyl ester, polymer with ethenol	25213-24-5	22.5%	19,905	55	75	<0.1	482
52	Inorganic potassium compound/alkali hydroxide	Proprietary	22.5%	--	--	--	--	--
53	Non-hazardous ingredients	Proprietary	22.2%	--	--	--	--	--
54	Mixture	Proprietary	22.1%	--	--	--	--	--
55	Limestone	1317-65-3	22.0%	448,313	1,456	1,718	6.2	11,113

Rank	Constituent	CASRN	Freq. of use (%)	Total mass (kg)	Median mass per event (kg)	Mean mass per event (kg)	Min mass per event (kg)	Max mass per event (kg)
56	Calcium carbonate	471-34-1	21.7%	2,719,556	9,319	10,541	0.6	147,567
57	Calcium chloride	10043-52-4	21.7%	7,194,553	115	27,994	0.7	121,864
58	Silica	7631-86-9	21.6%	843,816	2,098	3,296	<0.1	132,998
59	Aluminum oxide	1344-28-1	21.5%	235,968	624	925	<0.1	13,737
60	Iron oxide	1309-37-1	21.2%	43,396	102	172	<0.1	2,645
61	Mica	12001-26-2	21.2%	379,061	1,174	1,504	1.2	37,265
62	Wood chemicals	Proprietary	21.0%	--	--	--	--	--
63	Sulfur compound	Proprietary	20.8%	--	--	--	--	--
64	Salt compound	Proprietary	20.7%	--	--	--	--	--
65	Naphthalene sulfonate-formaldehyde condensate, sodium salt	Proprietary	20.3%	--	--	--	--	--
66	Salt of organic acid	Proprietary	20.2%	--	--	--	--	--
67	Carbon	7440-44-0	19.8%	35,749	107	152	0.1	2,643
68	Aliphatic alcohol	Proprietary	19.7%	--	--	--	--	--
69	Fatty acids ester	Proprietary	19.2%	--	--	--	--	--
70	Sodium carbonate	497-19-8	19.1%	94,121	227	415	<0.1	2,699
71	Modified sulfonate	Proprietary	19.0%	--	--	--	--	--
72	Fumes, silica	69012-64-2	18.9%	186,990	570	835	6.5	4,325
73	Quinoline	91-22-5	18.8%	22	0.1	0.1	<0.1	1.9
74	Trydymite	15468-32-3	18.1%	4,437	7.1	21	1.5	230
75	Saponite	1319-41-1	17.9%	835,598	2,722	3,923	90.7	63,299
76	Aromatic hydrocarbon A (7A)	Proprietary	17.1%	--	--	--	--	--
77	Aromatic hydrocarbon A (7B)	Proprietary	17.1%	--	--	--	--	--
78	Aromatic hydrocarbon A (7C)	Proprietary	17.1%	--	--	--	--	--
79	Sodium polyacrylate	Proprietary	16.8%	318,807	1,384	1,602	181	11,181
80	Aluminum	7429-90-5	16.5%	2,212	9.1	11	1.8	53
81	Acrylic polymer	203008-81-5	16.3%	17,095	79	88	20	603
82	Acetone	67-64-1	15.5%	26	0.1	0.1	<0.1	3.6
83	Welan gum	72121-88-1	15.3%	920	4.1	5.1	1.4	43
84	Detergent	Proprietary	14.3%	--	--	--	--	
85	Polysaccharide	Proprietary	14.3%	--	--	--	--	
86	Petroleum resins	64742-16-1	14.2%	281	1.3	1.7	0.3	8.0

Rank	Constituent	CASRN	Freq. of use (%)	Total mass (kg)	Median mass per event (kg)	Mean mass per event (kg)	Min mass per event (kg)	Max mass per event (kg)
87	Calcium bromide	7789-41-5	13.7%	774	0.7	4.7	0.2	610
88	Poly(oxy-1,2-ethandiyl), a-(nonylphenyl)-w-hydroxy-	9016-45-9	13.2%	965	4.6	6.1	0.2	35
89	Cellulose, microcrystalline	9004-34-6	13.1%	120,351	431	777	0.1	17,760
90	Copolymer	Proprietary	13.1%	--	--	--	--	--
91	Lubricant	Proprietary	12.8%	--	--	--	--	--
92	Stearic acid	57-11-4	12.1%	22,416	150	156	1.2	487
93	1,2-Ethanediaminium, N1,N2-bis[2-[bis(2-hydroxyethyl) methylammonio]ethyl]-N1,N2-bis(2-hydroxyethyl)-N1,N2-dimethyl-, chloride (1:4)	138879-94-4	11.5%	16,052	96	118	13	959
94	Sodium lignosulfate	8061-51-6	11.4%	1,440	10	11	<0.1	29
95	Sodium gluconate	527-07-1	11.1%	1,518	10	12	1.1	79
96	Aromatic hydrocarbon (7A)	Proprietary	10.4%	--	--	--	--	--
97	Aromatic hydrocarbon (7B)	Proprietary	10.4%	--	--	--	--	--
98	Ester	Proprietary	10.2%	--	--	--	--	--
99	Polymer	Proprietary	10.2%	20,676	103	225	15	3,492
100	Pine oil	8002-09-3	9.8%	607	4.5	5.2	<0.1	19
101	Aromatic amines	Proprietary	9.6%	15	15	15	15	15
102	Ethyloctynol	5877-42-9	9.6%	640	4.5	5.6	<0.1	51
103	Isoquinoline	119-65-3	9.6%	592	4.5	5.2	<0.1	19
104	Nonylphenol ethoxylate	Proprietary	9.6%	5.1	5.1	5.1	5.1	5.1
105	Quinaldine	91-63-4	9.5%	593	4.5	5.2	<0.1	19
106	Aromatic amine, TOFA salt	Proprietary	8.9%	9.1	9.1	9.1	9.1	9.1
107	Viscosifier	Proprietary	8.9%	--	--	--	--	--
108	Inorganic solvent	Proprietary	8.2%	--	--	--	--	--
109	P-mentha-1,8-diene	138-86-3	8.2%	19,230	179	198	1.0	691
110	Modified starch	Proprietary	7.9%	--	--	--	--	--
111	Magnesium	7439-95-4	7.5%	471,029	4,941	5,293	489	82,343
112	Acetic acid	64-19-7	7.4%	45,407	286	516	1.1	7,028
113	Organophosphonic acid salt	Proprietary	7.4%	--	--	--	--	--
114	Aldehyde	Proprietary	7.3%	--	--	--	--	--
115	Amide surfactant	Proprietary	7.2%	--	--	--	--	--

Rank	Constituent	CASRN	Freq. of use (%)	Total mass (kg)	Median mass per event (kg)	Mean mass per event (kg)	Min mass per event (kg)	Max mass per event (kg)
116	Amide surfactant phosphate acid salt	Proprietary	6.6%	--	--	--	--	--
117	Amide surfactant phosphate ester salt	Proprietary	6.5%	--	--	--	--	--
118	Anionic acrylamide copolymer	Proprietary	5.9%	100,992	1,452	1,554	476	4,173
119	Solvent naphtha, petroleum, light arom.	64742-95-6	5.8%	2,655	1.7	39	0.7	2,274
120	1,2,4-Trimethylbenzene	95-63-6	5.7%	1,231	1.6	18	0.7	819
121	Isopropanol	67-63-0	5.7%	797	5.3	12	<0.1	196
122	Tetrasodium pyrophosphate	7758-16-9	5.7%	7,764	68	114	<0.1	1,202
123	Cellophane	9005-81-6	5.6%	8,030	59	120	0.1	2,225
124	Cellulose, 2-hydroxyethyl ether	9004-62-0	5.5%	6,686	63	103	8.6	1,526
125	Cristobalite	14464-46-1	5.1%	28,771	289	472	61	6,495
126	Halides, inorganic salt	Proprietary	5.1%	--	--	--	--	--
127	Modified thiourea polymer	Proprietary	5.1%	--	--	--	--	--
128	Calcium magnesium oxide	37247-91-9	5.0%	13,246	100	225	16	6,761
129	Ionic surfactants	Proprietary	4.1%	--	--	--	--	--
130	2-Propenoic acid, homopolymer, sodium salt	9003-04-7	4.0%	13,909	133	296	<0.1	1,945
131	Formaldehyde, polymer with 2-methyloxirane, 4-nonylphenol and oxirane	63428-92-2	3.6%	295	1.7	6.9	1.6	66
132	Ionic surfactant	Proprietary	3.6%	--	--	--	--	--
133	Calcium hydroxide	1305-62-0	3.5%	42,250	136	1,031	2.5	12,156
134	Proprietary blend	Proprietary	3.4%	--	--	--	--	--
135	Naphthalene sulfonate-formaldehyde condensate, sodium salt	9084-06-4	3.0%	411	9.0	11	2.7	33
136	Polynuclear aromatic hydrocarbons	Proprietary	3.0%	--	--	--	--	--
137	Organo sulfur compounds	Proprietary	2.9%	--	--	--	--	--
138	Phosphoric acid	7664-38-2	2.9%	4,504	82	129	0.4	879
139	Tetrasodium pyrophosphate	7722-88-5	2.9%	7,080	159	202	64	431
140	Aromatic hydrocarbon (7)	Proprietary	2.9%	--	--	--	--	--
141	Nitrilotriacetic acid	139-13-9	2.9%	6,252	83	184	8.6	649

Rank	Constituent	CASRN	Freq. of use (%)	Total mass (kg)	Median mass per event (kg)	Mean mass per event (kg)	Min mass per event (kg)	Max mass per event (kg)
142	Ethanol	64-17-5	2.8%	298	8.6	9.0	1.2	29
143	Silanetriol, (3-aminopropyl)-, homopolymer	68400-07-7	2.7%	1,729	51	54	7.2	176
144	Silanetriol, 1-(3-aminopropyl)-	58160-99-9	2.7%	288	8.6	9.0	1.2	29
145	Aromatic compound	Proprietary	2.5%	--	--	--	--	--
146	Ethylene glycol	107-21-1	2.5%	81	0.5	2.7	<0.1	31
147	Ferrous sulfate	17375-41-6	2.4%	173	2.8	6.0	0.2	45
148	Polycyclic compound	Proprietary	2.4%	--	--	--	--	--
149	Polypropylene glycol	25322-69-4	2.3%	32	0.1	1.2	<0.1	4.0
150	Sulfuric acid	7664-93-9	2.1%	6.2	<0.1	0.2	<0.1	1.7
151	2-Ethylhexan-1-ol	104-76-7	2.0%	2,205	16	92	0.9	480
152	Bis(isopropyl)naphthalene	38640-62-9	2.0%	83	1.8	3.5	1.5	26
153	Calcium sulfate	7778-18-9	2.0%	419	13	18	0.1	48
154	Ashes (residues), coal	68131-74-8	1.9%	127,226	4,112	5,532	1,007	23,689
155	Isobutylmethylcarbinol	108-11-2	1.9%	57	1.8	2.5	1.5	12
156	Boric acid	10043-35-3	1.9%	31	0.9	1.4	<0.1	4.0
157	Glassy calcium magnesium phosphate	65997-17-3	1.9%	51,860	2,138	2,357	490	4,658
158	Petroleum resins	Proprietary	1.9%	--	--	--	--	--
159	Mineral fiber	Proprietary	1.8%	11,097	467	528	23	1,728
160	Mullite	1302-93-8	1.8%	14,867	641	708	147	1,398
161	Polyester	Proprietary	1.8%	56	3.2	3.1	2.6	3
162	Siloxanes and silicones, di-Me	67762-90-7	1.7%	79	<0.1	4.0	<0.1	79
163	Vegetable and polymer fibers	Proprietary	1.7%	563	563	563	563	563
164	Gilsonite	12002-43-6	1.6%	10657	363	561	22	2,654
165	Humic acid	1415-93-6	1.6%	10,171	516	535	90	1,168
166	Polysiloxanes, di-Me	63148-62-9	1.6%	0.1	<0.1	<0.1	<0.1	<0.1
167	Quaternary ammonium compound	Proprietary	1.5%	--	--	--	--	--
168	Quaternary ammonium compound (1)	Proprietary	1.5%	--	--	--	--	--
169	Quaternary ammonium compound (2)	Proprietary	1.5%	--	--	--	--	--
170	Quaternary ammonium compound (3)	Proprietary	1.5%	--	--	--	--	--

Rank	Constituent	CASRN	Freq. of use (%)	Total mass (kg)	Median mass per event (kg)	Mean mass per event (kg)	Min mass per event (kg)	Max mass per event (kg)
171	Dodecylbenzene sulfonic acid	27176-87-0	1.4%	95	<0.1	5.6	<0.1	94
172	Propylene glycol	57-55-6	1.4%	180	<0.1	11	<0.1	180
173	Quaternary ammonium compound-1	Proprietary	1.4%	--	--	--	--	--
174	Quaternary ammonium compound-2	Proprietary	1.4%	--	--	--	--	--
175	Quaternary ammonium compound-3	Proprietary	1.4%	--	--	--	--	--
176	Surfactant	Proprietary	1.4%	--	--	--	--	--
177	Toluene	108-88-3	1.4%	1,934	6.7	114	0.3	660
178	Fatty acids, tall oil, reaction products with triethanolamine	67784-78-5	1.3%	0.2	<0.1	<0.1	<0.1	<0.1
179	Fuller's earth	8031-18-3	1.3%	0.9	0.1	0.1	<0.1	0.1
180	Poly(oxy-1,2-ethanediyl), .alpha.-[bis(1-methylpropyl)phenyl]-.omega.-hydroxy-	53964-94-6	1.3%	0.4	<0.1	<0.1	<0.1	0.1
181	Polyethylene glycol monostearate	9004-99-3	1.3%	<0.1	<0.1	<0.1	<0.1	<0.1
182	Potassium acetate	127-08-2	1.3%	<0.1	<0.1	<0.1	<0.1	<0.1
183	Sorbic acid	110-44-1	1.3%	<0.1	<0.1	<0.1	<0.1	<0.1
184	Sorbitan monostearate	1338-41-6	1.3%	<0.1	<0.1	<0.1	<0.1	<0.1
185	Sulfuric acid, calcium salt, hydrate (2:2:1)	10034-76-1	1.3%	1,588	55	99	44	407
186	Urea	57-13-6	1.3%	4,677	31	292	0.1	2,404
187	Ethoxylated C14-15 alcohols	68951-67-7	1.3%	76	2.4	5.1	0.7	29.
188	Naphthalene sulfonic acid, bis(1-methylethyl)-, compd. with cyclohexanamine (1:1)	Proprietary	1.3%	--	--	--	--	--
189	Benzenesulfonic acid, C10-16-alkyl derivs., compds. with cyclohexylamine	Proprietary	1.2%	--	--	--	--	--
190	Ethoxylated alcohol	Proprietary	1.2%	--	--	--	--	--
191	Etidronic acid	2809-21-4	1.2%	8,001	543	572	240	1350
192	Methyl ester of sulfonate tannin	Proprietary	1.2%	795	50	72	13	274

Rank	Constituent	CASRN	Freq. of use (%)	Total mass (kg)	Median mass per event (kg)	Mean mass per event (kg)	Min mass per event (kg)	Max mass per event (kg)
193	Nonionic surfactants	Proprietary	1.2%	--	--	--	--	--
194	Phosphonic acid	13598-36-2	1.2%	2,400	163	171	72	405
195	Polyacrylate / polyacrylamide polymer blend	Proprietary	1.2%	113	7.3	8.7	0.9	30
196	Tricalcium phosphate	7758-87-4	1.2%	37	0.5	2.7	<0.1	30
197	Blend	Proprietary	1.1%	--	--	--	--	--
198	Non-hazardous	Proprietary	1.1%	--	--	--	--	--
199	Polypropylene	9003-07-0	1.1%	696	56	54	11	81
200	2-Propenoic acid, 2-methyl-, polymer with 2-propenoic acid	25751-21-7	1.0%	7.0	0.7	0.6	<0.1	0.8
201	Ethylene oxide	75-21-8	1.0%	0.5	<0.1	<0.1	<0.1	0.5
202	Formic acid	64-18-6	1.0%	2,497	40	208	12	1,159
203	Lignosulfonate	Proprietary	1.0%	--	--	--	--	--
204	Alkylaryl sulfonate	Proprietary	0.9%	--	--	--	--	--
205	Benzene, C10-C16 alkyl derivatives	68648-87-3	0.9%	0.7	<0.1	0.1	<0.1	0.2
206	Boron sodium oxide	1330-43-4	0.9%	1,074	98	98	98	98
207	Diutan	125005-87-0	0.9%	0.2	<0.1	<0.1	<0.1	<0.1
208	Mineral	Proprietary	0.9%	--	--	--	--	--
209	Olefins	Proprietary	0.9%	--	--	--	--	--
210	Oxylated alcohol	Proprietary	0.9%	--	--	--	--	--
211	Polyvinyl alcohol	9002-89-5	0.9%	618	55	56	35	80
212	Potassium oxide	12136-45-7	0.9%	1,241	85	113	59	250
213	Quaternary ammonium compound	61790-59-8	0.9%	3.2	<0.1	0.3	<0.1	3.2
214	Sodium formate	141-53-7	0.9%	68	5.6	6.2	0.2	23
215	1-Propanesulfonic acid, 2-methyl-2-[(1-oxo-2-propenyl)amino]-, monoammonium salt, polymer with 2-propenamide	110897-64-8	0.8%	1.5	0.1	0.1	0.1	0.2
216	Acrylamide	79-06-1	0.8%	<0.1	<0.1	<0.1	<0.1	<0.1
217	Amphoteric surfactant	Proprietary	0.8%	--	--	--	--	--
218	Aromatic hydrocarbon (3)	Proprietary	0.8%	--	--	--	--	--

Rank	Constituent	CASRN	Freq. of use (%)	Total mass (kg)	Median mass per event (kg)	Mean mass per event (kg)	Min mass per event (kg)	Max mass per event (kg)
219	Fatty acids, tall oil	Proprietary	0.8%	--	--	--	--	--
220	Oxide support	Proprietary	0.8%	--	--	--	--	--
221	Polyacrylamide	9003-05-8	0.8%	241	25	24	<0.1	48
222	Proprietary	Proprietary	0.8%	262	30	33	16	52
223	Salt of fatty acid polyamine	Proprietary	0.8%	--	--	--	--	--
224	Silica support	Proprietary	0.8%	--	--	--	--	--
225	Sodium aluminate	1302-42-7	0.8%	809	47	81	9.1	255
226	Sulfonic acid salt	Proprietary	0.8%					--
227	Tall oil	8002-26-4	0.8%	3,912	13	391	0.7	3,538
228	Aromatic hydrocarbon (4)	Proprietary	0.8%	--	--	--	--	--
229	Ethoxylated nonylphenol	Proprietary	0.8%	--	--	--	--	--
230	Organophilic clay	Proprietary	0.8%	--	--	--	--	--
231	Polyether	Proprietary	0.8%	184	14	20	1.1	60
232	Sodium acetate	127-09-3	0.8%	11	1.0	1.5	0.1	5.1
233	Sodium sulfate	7757-82-6	0.8%	291	2.4	32	<0.1	191
234	Synthetic acid	Proprietary	0.8%	--	--	--	--	--
235	2-Mercaptoethyl alcohol	60-24-2	0.7%	27	2.5	3.4	1.2	8.9
236	Alkyl aryl amine sulfonate	Proprietary	0.7%	--	--	--	--	--
237	Aromatic aldehyde	Proprietary	0.7%	--	--	--	--	--
238	Aromatic hydrocarbon (5)	Proprietary	0.7%	--	--	--	--	--
239	Borate	Proprietary	0.7%	--	--	--	--	--
240	Cocamidopropyl betaine	61789-40-0	0.7%	49	<0.1	6.2	<0.1	49
241	Compound synthetic diesters	8029-39-8	0.7%	240	19	30	9.1	84
242	Cyclic alkanes	Proprietary	0.7%	--	--	--	--	--
243	Cyclohexamine sulfate	19834-02-7	0.7%	2.0	0.1	0.2	0.1	0.9
244	Diethanolamine	111-42-2	0.7%	0.1	<0.1	<0.1	<0.1	<0.1
245	Ketone	Proprietary	0.7%	--	--	--	--	--
246	Linear alkylbenzene	Proprietary	0.7%	--	--	--	--	--
247	Oxyalkylated fatty acid	Proprietary	0.7%	--	--	--	--	--
248	Poly(oxy-1,2-ethanediyl), .alpha.-isodecyl-.omega.-hydroxy-, phosphate, potassium salt	68071-17-0	0.7%	29	0.6	3.6	0.3	15
249	Salt of inorganic acid	Proprietary	0.7%	--	--	--	--	--

Rank	Constituent	CASRN	Freq. of use (%)	Total mass (kg)	Median mass per event (kg)	Mean mass per event (kg)	Min mass per event (kg)	Max mass per event (kg)
250	Sodium oxide	12401-86-4	0.7%	939	91	117	59	250
251	Sodium silicate	1344-09-8	0.7%	5,721	72	715	34	2,666
252	Strontium chloride	10476-85-4	0.7%	194	23	24	2.3	48
253	Unsaturated alcohol	Proprietary	0.7%	--	--	--	--	--
254	Wood dust, soft wood	Proprietary	0.7%	1,349	68	169	45	674
255	2-Butenedioic acid (E)-, polymer with 1,2-ethanediol and .alpha.,.alpha.?-[(1-methylethylidene)di-4,1-phenylene]bis[.omega.-hydroxypoly[oxy(methyl-1,2-ethanediyl)]]	39382-21-3	0.6%	<0.1	<0.1	<0.1	<0.1	<0.1
256	4,7-Methano-1H-indene, 3A,4,7,7A-tetrahydro-, polymer with 1,3-cyclopentadiene, cyclopentene, 1-hexene, 2-methyl-2-butene and 1,3-pentadiene	68003-51-0	0.6%	<0.1	<0.1	<0.1	<0.1	<0.1
257	Alcohols, C10-14, ethoxylated	66455-15-0	0.6%	456	64	65	28	107
258	Ammonium hydroxide	1336-21-6	0.6%	<0.1	<0.1	<0.1	<0.1	<0.1
259	Cellulose derivative	Proprietary	0.6%	181	91	91	45	136
260	Coal, <5% SiO2	Proprietary	0.6%	9,081	1,057	1,297	420	3,157
261	Esters of rosin oligomers with pentaerythritol	65997-12-8	0.6%	<0.1	<0.1	<0.1	<0.1	<0.1
262	Ethanesulfonic acid, 2-[methyl[(9Z)-1-oxo-9-octadecen-1-Yl]amino]-, sodium salt (1:1)	137-20-2	0.6%	4,995	53	714	6.1	2,881
263	Morpholine	110-91-8	0.6%	38	5.4	5.4	2.4	8.9
264	Mutual solvent	Proprietary	0.6%	--	--	--	--	--
265	Nonionic surfactant	Proprietary	0.6%	93	<0.1	13	<0.1	80
266	Organic sulfonic acid amine salt	Proprietary	0.6%	--	--	--	--	--
267	Silicon dioxide crystalline	60676-86-0	0.6%	5.3	0.7	0.8	0.2	1.2
268	Alkylene oxide block polymer	Proprietary	0.5%	--	--	--	--	--
269	Biopolymer	Proprietary	0.5%	--	--	--	--	--

Rank	Constituent	CASRN	Freq. of use (%)	Total mass (kg)	Median mass per event (kg)	Mean mass per event (kg)	Min mass per event (kg)	Max mass per event (kg)
270	Diethylene glycol	111-46-6	0.5%	4.2	0.5	0.7	0.2	1.8
271	Ferric chloride	7705-08-0	0.5%	171	30	29	3.5	52
272	Potassium hydroxide	1310-58-3	0.5%	4,198	330	700	<0.1	2,495
273	Sodium hydroxide	1310-73-2	0.5%	28	<0.1	4.7	<0.1	15
274	Tannin, sodium salt polymer with acrylic monomers	Proprietary	0.5%	--	--	--	--	--
275	Walnut shells	Proprietary	0.5%	3,538	488	590	113	1,111
276	Water (including mix water supplied by client)	Proprietary	0.5%	159,143	27,193	26,524	24,310	27,540
277	1-Eicosene	3452-07-1	0.4%	<0.1	<0.1	<0.1	<0.1	<0.1
278	1-Hexadecene	629-73-2	0.4%	0.9	0.2	0.2	0.1	0.2
279	1-Octadecene	112-88-9	0.4%	0.9	0.2	0.2	0.1	0.2
280	1-Tetradecene	1120-36-1	0.4%	<0.1	<0.1	<0.1	<0.1	<0.1
281	Acrylic co-polymer	Proprietary	0.4%	--	--	--	--	--
282	Amide surfactant phosphate	Proprietary	0.4%	--	--	--	--	--
283	Fatty acids, tall oil	61790-12-3	0.4%	31	7.1	6.3	2.4	8.6
284	Glycol	Proprietary	0.4%	--	--	--	--	--
285	Halad-322	Proprietary	0.4%	--	--	--	--	--
286	Modified lignosulfonate	Proprietary	0.4%	--	--	--	--	--
287	Naphthalene sulfonic acid, polymer with formaldehyde, sodium salt	Proprietary	0.4%	45	9.0	9.0	9.0	9.0
288	Silicone	Proprietary	0.4%	--	--	--	--	--
289	Silicone fluid	Proprietary	0.4%	--	--	--	--	--
290	1-Butanol	71-36-3	0.3%	<0.1	<0.1	<0.1	<0.1	<0.1
291	1-Propanaminium, N-(carboxymethyl)-N,N-dimethyl-3-(((13Z)-1-oxo-13-docosenyl)amino)-, inner salt	149879-98-1	0.3%	13	3.2	3.2	2.3	4.3
292	Acetic acid ethenyl ester, polymer with choroethene and ethene	25085-46-5	0.3%	<0.1	<0.1	<0.1	<0.1	<0.1
293	Acetophenone	98-86-2	0.3%	3.9	<0.1	1.0	<0.1	3.9
294	Amide surfactant ester acid salt	Proprietary	0.3%	--	--	--	--	--
295	Ammonium fluoride	12125-01-8	0.3%	1,141	272	285	261	336

Rank	Constituent	CASRN	Freq. of use (%)	Total mass (kg)	Median mass per event (kg)	Mean mass per event (kg)	Min mass per event (kg)	Max mass per event (kg)
296	Anti-foamer	Proprietary	0.3%	--	--	--	--	--
297	Benzenesulfonic acid, C10-16-alkyl derivs., compds. with 2-propanamine	68584-24-7	0.3%	4.5	1.1	1.1	0.5	1.9
298	Benzenesulfonic acid, C10-16-alkyl derivs., compds. with triethanolamine	68584-25-8	0.3%	4.5	1.1	1.1	0.5	1.9
299	Carbon	Proprietary	0.3%	--	--	--	--	--
300	Coal, ground	50815-10-6	0.3%	429	94	107	15	227
301	Corrosion inhibitor	Proprietary	0.3%	--	--	--	--	--
302	Dispersant	Proprietary	0.3%	--	--	--	--	--
303	Emulsion of complex stearates	Proprietary	0.3%	102	23	26	23	34
304	Ethoxylated hexanol	68439-45-2	0.3%	58	16	15	3.7	22
305	Fatty acid ester	Proprietary	0.3%	--	--	--	--	--
306	Formate salt	Proprietary	0.3%	--	--	--	--	--
307	Graphite	7782-42-5	0.3%	704	101	176	7.5	494
308	Inorganic potassium	Proprietary	0.3%	--	--	--	--	--
309	Lignosulfonic acid, calcium salt	8061-52-7	0.3%	0.1	<0.1	<0.1	<0.1	0.1
310	Linear/branched alcohol ethoxylate (11eo)	127036-24-2	0.3%	<0.1	<0.1	<0.1	<0.1	<0.1
311	Petroleum distillates	68990-35-2	0.3%	109	24	27	22	39
312	Poly(sodium styrenesulfonate)	25704-18-1	0.3%	<0.1	<0.1	<0.1	<0.1	<0.1
313	Silica fume	Proprietary	0.3%	--	--	--	--	--
314	Sodium chloroacetate	3926-62-3	0.3%	<0.1	<0.1	<0.1	<0.1	<0.1
315	Sulfonate salt	Proprietary	0.3%	--	--	--	--	--
316	Sulfuric acid, barium salt (1:1)	Proprietary	0.3%	3,311	726	828	680	1,179
317	Talc	14807-96-6	0.3%	<0.1	<0.1	<0.1	<0.1	<0.1
318	Undecanol, branched and linear	128973-77-3	0.3%	<0.1	<0.1	<0.1	<0.1	<0.1
319	Vinylidene chloride/methylacrylate copolymer	25038-72-6	0.3%	<0.1	<0.1	<0.1	<0.1	<0.1
320	1,2,3-Trimethylbenzene	526-73-8	0.3%	3.2	1.0	1.1	0.9	1.4
321	1,3,5-Trimethylbenzene	108-67-8	0.3%	7.3	2.3	2.4	1.9	3.1
322	Acid modified petroleum residue	Proprietary	0.3%	--	--	--	--	--

Rank	Constituent	CASRN	Freq. of use (%)	Total mass (kg)	Median mass per event (kg)	Mean mass per event (kg)	Min mass per event (kg)	Max mass per event (kg)
323	Aliphatic alcohol (1)	Proprietary	0.3%	--	--	--	--	--
324	Aliphatic alcohol (2)	Proprietary	0.3%	--	--	--	--	--
325	Aliphatic alcohol (3)	Proprietary	0.3%	--	--	--	--	--
326	Alkyl benzenesulfonic acid	Proprietary	0.3%	--	--	--	--	--
327	Alkyl diamide	Proprietary	0.3%	--	--	--	--	--
328	Alkyne alcohol	Proprietary	0.3%	--	--	--	--	--
329	Ammonium salt	Proprietary	0.3%	--	--	--	--	--
330	Ammonium sulfate	7783-20-2	0.3%	21.6	7.2	7.2	7.2	7.2
331	Anionic polyacrylamide	Proprietary	0.3%	50	13	17	13	25
332	Anionic polymer	Proprietary	0.3%	--	--	--	--	--
333	Aromatic amines, TOFA salt	Proprietary	0.3%	--	--	--	--	--
334	Aromatic compound (1)	Proprietary	0.3%	--	--	--	--	--
335	Aromatic compound (2)	Proprietary	0.3%	--	--	--	--	--
336	Aromatic compound (3)	Proprietary	0.3%	--	--	--	--	--
337	Aromatic compound (4)	Proprietary	0.3%	--	--	--	--	--
338	Aromatic compound (5)	Proprietary	0.3%	--	--	--	--	--
339	Aromatic compound (6)	Proprietary	0.3%	--	--	--	--	--
340	Aromatic hydrocarbon	Proprietary	0.3%	--	--	--	--	--
341	Canola oil	120962-03-0	0.3%	350	92	117	75	183
342	Cement retarder	Proprietary	0.3%	--	--	--	--	--
343	Clay	Proprietary	0.3%	--	--	--	--	--
344	Complex stearates	Proprietary	0.3%	91	23	30	11	57
345	Cyclohexasiloxane, 2,2,4,4,6,6, 8,8,10,10,12,12-dodecamethyl-	540-97-6	0.3%	0.1	<0.1	<0.1	<0.1	<0.1
346	Cyclopentasiloxane, 2,2,4,4,6,6, 8,8,10,10-decamethyl-	541-02-6	0.3%	0.1	<0.1	<0.1	<0.1	<0.1
347	DBNPA (2,2-dibromo-3-nitrilopropionamide)	10222-01-2	0.3%	10	4.1	3.3	<0.1	5.9
348	Enviro Og	Proprietary	0.3%	--	--	--	--	--
349	Fatty acid	Proprietary	0.3%	--	--	--	--	--
350	Formaldehyde, polymer with 4-(1,1-dimethylethyl)phenol, 2-methyloxirane and oxirane	30704-64-4	0.3%	0.1	<0.1	<0.1	<0.1	0.1
351	Guar gum	9000-30-0	0.3%	1,667	819	556	2.6	845

Rank	Constituent	CASRN	Freq. of use (%)	Total mass (kg)	Median mass per event (kg)	Mean mass per event (kg)	Min mass per event (kg)	Max mass per event (kg)
352	Inorganic salt of an acid	Proprietary	0.3%	--	--	--	--	--
353	Ionic compound	Proprietary	0.3%	--	--	--	--	--
354	Lecithins	8002-43-5	0.3%	4.7	1.4	1.6	0.3	3.0
355	MBNPA (2-bromo-3-nitrilopropionamide)	1113-55-9	0.3%	<0.1	<0.1	<0.1	<0.1	<0.1
356	Monoethanolamine borate (1:X)	26038-87-9	0.3%	245	123	82	0.1	123
357	Nap-formaldehyde condensate, sodium salt	Proprietary	0.3%	--	--	--	--	--
358	Naphthalene sulfonate-formaldehyde	Proprietary	0.3%	--	--	--	--	--
359	Nonionic defoaming agent	Proprietary	0.3%	--	--	--	--	--
360	Octamethylcyclotetrasiloxane	556-67-2	0.3%	0.1	<0.1	<0.1	<0.1	<0.1
361	Organosulfur compound (1)	Proprietary	0.3%	--	--	--	--	--
362	Organosulfur compound (2)	Proprietary	0.3%	--	--	--	--	--
363	Oxyalkylated polyamine	Proprietary	0.3%	--	--	--	--	--
364	Phosphonate salt	Proprietary	0.3%	--	--	--	--	--
365	Polyacrylate	Proprietary	0.3%	--	--	--	--	--
366	Polyanionic cellulosic polymer	Proprietary	0.3%	--	--	--	--	--
367	Polyanionic polymer	Proprietary	0.3%	--	--	--	--	--
368	Polycyclic aromatic hydrocarbon	Proprietary	0.3%	--	--	--	--	--
369	Sodium persulfate	7775-27-1	0.3%	35	16	12	<0.1	19
370	Sugar	Proprietary	0.3%	98	34	33	9.1	54
371	Sulfate	Proprietary	0.3%	--	--	--	--	--
372	Tetrasodium ethylenediaminetetraacetate	64-02-8	0.3%	0.1	<0.1	<0.1	<0.1	<0.1
373	Vinyl copolymer	Proprietary	0.3%	--	--	--	--	--
374	2-Methyl-3(2H)-isothiazolone	2682-20-4	0.2%	5.2	2.6	2.6	<0.1	5.2
375	Acetic anhydride	108-24-7	0.2%	49	25	25	8.2	41
376	Aliphatic sulfonate	Proprietary	0.2%	--	--	--	--	--
377	Aluminum oxide silicate	12141-46-7	0.2%	140	70	70	31	110
378	Amide surfactant salt of organic acid	Proprietary	0.2%	--	--	--	--	--
379	Amine salts	Proprietary	0.2%	--	--	--	--	--

Rank	Constituent	CASRN	Freq. of use (%)	Total mass (kg)	Median mass per event (kg)	Mean mass per event (kg)	Min mass per event (kg)	Max mass per event (kg)
380	Blend of vegetable and polymer fibers	Proprietary	0.2%	--	--	--	--	--
381	D-Air 5000	Proprietary	0.2%	--	--	--	--	--
382	D-Mulse	Proprietary	0.2%	--	--	--	--	--
383	Enviro C-Inhib	Proprietary	0.2%	--	--	--	--	--
384	Enviro D-Mulse	Proprietary	0.2%	--	--	--	--	--
385	Ethoxylated alcohols C12-16	Proprietary	0.2%	--	--	--	--	--
386	Ethoxylated nonylphenol alcohol	Proprietary	0.2%	--	--	--	--	--
387	Ethoxylated surfactant	Proprietary	0.2%	--	--	--	--	--
388	Ethylene oxide-nonylphenol polymer	Proprietary	0.2%	--	--	--	--	--
389	Fatty acid esters	Proprietary	0.2%	--	--	--	--	--
390	Fatty acid oxyalkylate	Proprietary	0.2%	--	--	--	--	--
391	Glycerol	56-81-5	0.2%	0.3	0.1	0.1	0.1	0.2
392	HR-5	Proprietary	0.2%	--	--	--	--	--
393	Hydroxide	Proprietary	0.2%	--	--	--	--	--
394	Inorganic salt	Proprietary	0.2%	--	--	--	--	--
395	Lignin	9005-53-2	0.2%	0.2	0.1	0.1	0.1	0.1
396	Lithium carbonate	554-13-2	0.2%	43	22	22	11	32
397	Lithium chloride	7447-41-8	0.2%	43	22	22	11	32
398	Lithium hydroxide	1310-65-2	0.2%	43	22	22	11	32
399	Lithium hypochlorite	13840-33-0	0.2%	258	129	129	67	191
400	Modified acrylamide co-polymer	Proprietary	0.2%	--	--	--	--	--
401	M-SOLV	Proprietary	0.2%	--	--	--	--	--
402	Mud Flush III	Proprietary	0.2%	--	--	--	--	--
403	N,N-Bis(carboxymethyl)-L-glutamic acid	58976-65-1	0.2%	641	321	321	321	321
404	Naphtha (petroleum), heavy catalytic reformed	64741-68-0	0.2%	35	18	18	0.5	35
405	Naphthalene sulfonate-formaldehyde condensate, sodium salt	9008-63-3	0.2%	0.1	0.1	0.1	<0.1	0.1
406	Natural peat	Proprietary	0.2%	1,073	536	536	352	721

Rank	Constituent	CASRN	Freq. of use (%)	Total mass (kg)	Median mass per event (kg)	Mean mass per event (kg)	Min mass per event (kg)	Max mass per event (kg)
407	Organic acid salt 2	Proprietary	0.2%	--	--	--	--	--
408	Plaster of paris	26499-65-0	0.2%	1,394	697	697	697	697
409	Polyacrylamide	26006-22-4	0.2%	57	28	28	7.1	50
410	Polyacrylamide blend	Proprietary	0.2%	9.1	4.5	4.5	3.6	5.4
411	Polyphosphate ester	Proprietary	0.2%	1,436	718	718	25.9	1,410
412	Potassium hydrogen sulfate	7646-93-7	0.2%	86	43	43	22.5	64
413	Proprietary materials	Proprietary	0.2%	1,678	839	839	272	1,406
414	Quaternary amine	Proprietary	0.2%	--	--	--	--	--
415	Quaternary ammonium compounds, benzyl(hydrogenated tallow alkyl)dimethyl, stearates, salts with bentonite	121888-68-4	0.2%	4.2	2.1	2.1	2.1	2.1
416	Saturated alcohols	Proprietary	0.2%	--	--	--	--	--
417	Sawdust	Proprietary	0.2%	1,474	737	737	590	885
418	Silica	61790-53-2	0.2%	16,520	8,260	8,260	5,414	11,107
419	Silica gel	112926-00-8	0.2%	0.2	0.1	0.1	0.1	0.1
420	Sodium hypochlorite	7681-52-9	0.2%	4.7	2.3	2.3	2.3	2.3
421	Sulfurous acid, sodium salt (1:1), polymer with formaldehyde and 1,3,5-triazine-2,4,6-triamine	64787-97-9	0.2%	0.5	0.3	0.3	0.1	0.4
422	Surfactant mixture	Proprietary	0.2%	--	--	--	--	--
423	Surfactant, phosphate acid salt	Proprietary	0.2%	--	--	--	--	--
424	Tetrasodium glutamate diacetate	51981-21-6	0.2%	321	160	160	160	160
425	UCS	Proprietary	0.2%	--	--	--	--	--
426	Unknown	Proprietary	0.2%	--	--	--	--	--
427	Wood fiber	Proprietary	0.2%	1,402	701	701	27	1,374
428	Zinc sulfate	7733-02-0	0.2%	100	50	50	50	50
429	1-Methoxy-2-hydroxypropane	107-98-2	0.1%	94	94	94	94	94
430	1-Octanesulfonic acid sodium salt	5324-84-5	0.1%	157	157	157	157	157

Rank	Constituent	CASRN	Freq. of use (%)	Total mass (kg)	Median mass per event (kg)	Mean mass per event (kg)	Min mass per event (kg)	Max mass per event (kg)
431	2-Propen-1-aminium, N,N-dimethyl-N-2-propen-1-Yl-, chloride (1:1), homopolymer	26062-79-3	0.1%	75	75	75	75	75
432	2,4-Dihydroxy-2-methylpentane	107-41-5	0.1%	148	148	148	148	148
433	2-Propenoic acid, polymer with 2-propenamide	9003-06-9	0.1%	0.2	0.2	0.2	0.2	0.2
434	2-Propenoic acid, polymer with sodium 2-propenoate	9033-79-8	0.1%	10	10	10	10	10
435	5-Chloro-2-methyl-3(2H)-isothiazolone	26172-55-4	0.1%	5.2	5.2	5.2	5.2	5.2
436	Alcohol	Proprietary	0.1%	--	--	--	--	--
437	Alkenes	Proprietary	0.1%	--	--	--	--	--
438	Alkyl ether of corn sugar	Proprietary	0.1%	115	115	115	115	115
439	Aluminium distearate	300-92-5	0.1%	<0.1	<0.1	<0.1	<0.1	<0.1
440	Amide surfactant	68647-77-8	0.1%	292	292	292	292	292
441	Anionic copolymer	Proprietary	0.1%	--	--	--	--	--
442	Aromatic amines, TOFA salts	Proprietary	0.1%	--	--	--	--	--
443	Aromatic hydrocarbon (1) benzenesulfonic acid, C10-16-alkyl derivs., compds. with cyclohexylamine	Proprietary	0.1%	--	--	--	--	--
444	Aromatic hydrocarbons (7)	Proprietary	0.1%	--	--	--	--	--
445	BA-10A	Proprietary	0.1%	--	--	--	--	--
446	Benzene, tetrapropylene	25265-78-5	0.1%	2.7	2.7	2.7	2.7	2.7
447	Benzoisothiazolinone	2634-33-5	0.1%	<0.1	<0.1	<0.1	<0.1	<0.1
448	Calcium carbonate	Proprietary	0.1%	--	--	--	--	--
449	Calcium salts	Proprietary	0.1%	--	--	--	--	--
450	Calcium sulfate	Proprietary	0.1%	--	--	--	--	--
451	Cap	Proprietary	0.1%	--	--	--	--	--
452	Cellullose	Proprietary	0.1%	--	--	--	--	--
453	Cf Desco II	Proprietary	0.1%	--	--	--	--	--
454	Choline chloride	67-48-1	0.1%	141	141	141	141	141
455	C-Inhib	Proprietary	0.1%	--	--	--	--	--
456	Citrus extract	Proprietary	0.1%	57	57	57	57	57
457	Conqor 404	Proprietary	0.1%	--	--	--	--	--

Rank	Constituent	CASRN	Freq. of use (%)	Total mass (kg)	Median mass per event (kg)	Mean mass per event (kg)	Min mass per event (kg)	Max mass per event (kg)
458	Corrosion control	Proprietary	0.1%	--	--	--	--	--
459	Deflocculant	Proprietary	0.1%	--	--	--	--	--
460	Defoamer	Proprietary	0.1%	--	--	--	--	--
461	Defoamer 7	Proprietary	0.1%	--	--	--	--	--
462	Defoam-X	Proprietary	0.1%	--	--	--	--	--
463	Diethanolamine	Proprietary	0.1%	--	--	--	--	--
464	Diisopropylnaphthalenesulfonic acid	28757-00-8	0.1%	26	26	26	26	26
465	Distillates (petroleum), hydrotreated light; kerosene - unspecified	Proprietary	0.1%	57	57	57	57	57
466	Dodecyl(2-hydroxy-3-sulfonatopropyl)dimethylammonium	13197-76-7	0.1%	73	73	73	73	73
467	Dodecylbenzene	123-01-3	0.1%	5.4	5.4	5.4	5.4	5.4
468	Drilling detergent	Proprietary	0.1%	--	--	--	--	--
469	Drilling mud additive	Proprietary	0.1%	--	--	--	--	--
470	EDTA/copper chelate	Proprietary	0.1%	--	--	--	--	--
471	Emulsifier	Proprietary	0.1%	--	--	--	--	--
472	Enviro M-Solv	Proprietary	0.1%	--	--	--	--	--
473	Ethanol,2,2’-oxybis-,reaction products with ammonia, morpholine derivs., residues	68909-77-3	0.1%	91	91	91	91	91
474	Ethoxylate	Proprietary	0.1%	--	--	--	--	--
475	Ethoxylated alkyl amines	Proprietary	0.1%	--	--	--	--	--
476	Ethoxylated cyclic amines	Proprietary	0.1%	--	--	--	--	--
477	Glycolic acid	79-14-1	0.1%	89	89	89	89	89
478	Halad-344	Proprietary	0.1%	--	--	--	--	--
479	Inorganic nitrate salt	Proprietary	0.1%	--	--	--	--	--
480	Inorganic solvents	Proprietary	0.1%	--	--	--	--	--
481	Lignosulfonic acid, ethoxylated, sodium salts	68611-14-3	0.1%	543	543	543	543	543
482	Lithium chlorate	13453-71-9	0.1%	11	11	11	11	11
483	Lithium chlorate	Proprietary	0.1%	32	32	32	32	32
484	Lubricant (complex stearates)	Proprietary	0.1%	--	--	--	--	--

Rank	Constituent	CASRN	Freq. of use (%)	Total mass (kg)	Median mass per event (kg)	Mean mass per event (kg)	Min mass per event (kg)	Max mass per event (kg)
485	Maltodextrin	9050-36-6	0.1%	31	31	31	31	31
486	Mixture of surfactants	Proprietary	0.1%	--	--	--	--	--
487	Modified alkanolamide	Proprietary	0.1%	286	286	286	286	286
488	Modified lignin	Proprietary	0.1%	0.2	0.2	0.2	0.2	0.2
489	Monoethanolamine	141-43-5	0.1%	0.5	0.5	0.5	0.5	0.5
490	Naphthalene sulfonate-formaldehyde condensate	Proprietary	0.1%	--	--	--	--	--
491	Non-hazardous organic compound	Proprietary	0.1%	--	--	--	--	--
492	Nonylphenol	Proprietary	0.1%	--	--	--	--	--
493	Organic acid	Proprietary	0.1%	--	--	--	--	--
494	Petroleum distillate-mineral oil grade	8002-05-9	0.1%	30	30	30	30	30
495	Petroleum distillates	64741-44-2	0.1%	138,679	138,679	138,679	138,679	138679
496	Petroleum distillates	64742-46-7	0.1%	138,679	138,679	138,679	138,679	138679
497	Phosphate ester	Proprietary	0.1%	1,073	1,073	1,073	1,073	1073
498	Poly(oxy-1,2-ethanediyl), .alpha.-sulfo-.omega.-hydroxy-, C6-10-alkyl ethers, ammonium salts	68037-05-8	0.1%	297	297	297	297	297
499	Poly(oxy-1,2-ethanediyl), alpha-hexyl-omega-hydroxy	31726-34-8	0.1%	21	21	21	21	21
500	Polyepichlorohydrin, trimethyl amine quaternized	51838-31-4	0.1%	60	60	60	60	60
501	Polymer and water	Proprietary	0.1%	--	--	--	--	--
502	Polynuclear aromatic hydrocarbon	Proprietary	0.1%	--	--	--	--	--
503	Polyolphospate ester	Proprietary	0.1%	58	58	58	58	58
504	Potassium iodide	7681-11-0	0.1%	45	45	45	45	45
505	Powervis	Proprietary	0.1%	--	--	--	--	--
506	Proprietary blend of complex stearates	Proprietary	0.1%	791	791	791	791	791
507	Pumice	1332-09-8	0.1%	599	599	599	599	599
508	RCI 07289 corrosion inhibitor	Proprietary	0.1%	--	--	--	--	--
509	SA-1015	Proprietary	0.1%	--	--	--	--	--

Rank	Constituent	CASRN	Freq. of use (%)	Total mass (kg)	Median mass per event (kg)	Mean mass per event (kg)	Min mass per event (kg)	Max mass per event (kg)
510	Salt of amine/carbonyl condensate	Proprietary	0.1%	--	--	--	--	--
511	Silica sand gravel	Proprietary	0.1%	6,350	6,350	6,350	6,350	6350
512	Silica, crystalline, quartz	Proprietary	0.1%	15	15	15	15	15
513	Sodium borosilicate	50815-87-7	0.1%	1,361	1,361	1,361	1,361	1,361
514	Sodium polyacrylate/ polycrylamide	Proprietary	0.1%	--	--	--	--	--
515	Sodium salt	Proprietary	0.1%	--	--	--	--	--
516	Ss 26 in xylene	Proprietary	0.1%	--	--	--	--	--
517	Ssp-40 in xylene	Proprietary	0.1%	--	--	--	--	--
518	Starch	9005-25-8	0.1%	522	522	522	522	522
519	Sulfonated polymer	Proprietary	0.1%	--	--	--	--	--
520	Sulfonic acids, C14-16-alkane hydroxy and C14-16-alkene, sodium salts	68439-57-6	0.1%	5.4	5.4	5.4	5.4	5.4
521	Surfactant blend	Proprietary	0.1%	--	--	--	--	--
522	Thioglycolic acid	68-11-1	0.1%	98	98	98	98	98
523	Thrutrol	Proprietary	0.1%	--	--	--	--	--
524	Tuned Spacer III	Proprietary	0.1%	--	--	--	--	--
525	Wood dust	Proprietary	0.1%	163	163	163	163	163
