# Supplementary material for: Comparison of chemical-use between hydraulic fracturing, acidizing, and routine oil and gas development
Source: PLoS One. 2017 Apr 19;12(4):e0175344. doi: 10.1371/journal.pone.0175344 (PMC5396893; doi:10.1371/journal.pone.0175344)
Supplement: S2 Table — Total number of events is 1,187. (PDF) [file pone.0175344.s002.pdf]

S2 Table. The top 10% median masses of additives used in routine oil and gas development activities (exclusive of well stimulation) in the SCAQMD, June 4, 2013 to September 2, 2015. Total number of events is 1,187.

Constituent	CASRN	Freq. of use (%)	Total mass (kg)	Median mass per event (kg)	Mean mass per event (kg)	Min mass per event (kg)	Max mass per event (kg)
Petroleum distillates	64741-44-2	0.1%	138,679	138,679	138,679	138,679	138,679
Petroleum distillates	64742-46-7	0.1%	138,679	138,679	138,679	138,679	138,679
Water base fluid	7732-18-5	97.6%	196,128,245	48,117	169,514	-	4,483,250
Water (including mix water supplied by client)	Proprietary	0.5%	159,143	27,193	26,524	24,310	27,540
Portland cement	65997-15-1	24.9%	7,427,597	22,481	25,178	93	106,583
Barium sulfate	7727-43-7	23.9%	7,639,223	20,544	26,899	14	540,586
Calcium carbonate	471-34-1	21.7%	2,719,556	9,319	10,541	1	147,567
Silica	61790-53-2	0.2%	16,520	8,260	8,260	5,414	11,107
Crystalline silica quartz	14808-60-7	40.1%	3,917,132	7,165	8,229	7	65,385
Potassium chloride	7447-40-7	40.2%	28,907,629	7,035	60,603	0	1,136,781
Silica sand gravel	Proprietary	0.1%	6,350	6,350	6,350	6,350	6,350
Magnesium	7439-95-4	7.5%	471,029	4,941	5,292	489	82,343
Ashes (residues), coal	68131-74-8	1.9%	127,226	4,112	5,532	1,007	23,689
Gypsum	13397-24-5	24.3%	310,276,445	2,771	1,073,621	9	309,126,252
Saponite	1319-41-1	17.9%	835,598	2,722	3,923	91	63,299
Glassy calcium magnesium phosphate	65997-17-3	1.9%	51,860	2,138	2,357	490	4,658
Silica	7631-86-9	21.6%	843,816	2,098	3,296	0	132,997
Sulfonate	Proprietary	23.2%	500,337	1,746	1,939	68	10,047
Magnesium oxide	1309-48-4	24.3%	445,681	1,474	1,542	2	7,230
Limestone	1317-65-3	22.0%	448,313	1,456	1,718	6	11,113
Anionic acrylamide copolymer	Proprietary	5.9%	100,992	1,451	1,554	476	4,173
Calcium oxide	1305-78-8	24.4%	440,651	1,433	1,519	5	7,413
Sodium polyacrylate	Proprietary	16.8%	318,807	1,383	1,602	181	11,181
Sodium borosilicate	50815-87-7	0.1%	1,361	1,361	1,361	1,361	1,361
Hydrochloric acid	7647-01-0	54.8%	1,165,757	1,311	1,791	10	12,052
Mica	12001-26-2	21.2%	379,060	1,174	1,504	1	37,265
Phosphate ester	Proprietary	0.1%	1,073	1,073	1,073	1,073	1,073
Coal, <5% SiO2	Proprietary	0.6%	9,081	1,057	1,297	420	3,157
Cellulose, carboxymethyl ether, sodium salt	9004-32-4	22.7%	287,308	953	1,068	23	6,622

Constituent	CASRN	Freq. of use (%)	Total mass (kg)	Median mass per event (kg)	Mean mass per event (kg)	Min mass per event (kg)	Max mass per event (kg)
Proprietary materials	Proprietary	0.2%	1,678	839	839	272	1,406
Guar gum	9000-30-0	0.3%	1,667	819	556	3	845
Proprietary blend of complex stearates	Proprietary	0.1%	791	791	791	791	791
Sawdust	Proprietary	0.2%	1,474	737	737	590	885
Sulfuric acid, barium salt (1:1)	Proprietary	0.3%	3,311	726	828	680	1,179
Polyphosphate ester	Proprietary	0.2%	1,436	718	718	26	1,410
Wood fiber	Proprietary	0.2%	1,402	701	701	27	1,374
Plaster of paris	26499-65-0	0.2%	1,394	697	697	697	697
Mullite	1302-93-8	1.8%	14,867	641	708	147	1,398
Aluminum oxide	1344-28-1	21.5%	235,967	624	925	0	13,737
Pumice	1332-09-8	0.1%	599	599	599	599	599
Bentonite	1302-78-9	24.8%	708,757	570	2,427	7	217,271
Fumes, silica	69012-64-2	18.9%	186,990	570	835	6	4,325
Vegetable and polymer fibers	Proprietary	1.7%	562	562	562	562	562
Etidronic acid	2809-21-4	1.2%	8,001	543	571	240	1,350
Lignosulfonic acid, ethoxylated, sodium salts	68611-14-3	0.1%	543	543	543	543	543
Natural peat	Proprietary	0.2%	1,073	536	536	352	721
Starch	9005-25-8	0.1%	522	522	522	522	522
Humic acid	1415-93-6	1.6%	10,171	516	535	90	1,168
Walnut shells	Proprietary	0.5%	3,538	488	590	113	1,111
Mineral fiber	Proprietary	1.8%	11,097	467	528	23	1,728
Ammonium chloride	12125-02-9	48.4%	421,116	454	732	<1	18,847
Cellulose, microcrystalline	9004-34-6	13.1%	120,351	431	776	<1	17,760
Disodium metasilicate	6834-92-0	23.4%	93,416	367	336	<1	10,135
