# Supplementary material for: Comparison of chemical-use between hydraulic fracturing, acidizing, and routine oil and gas development
Source: PLoS One. 2017 Apr 19;12(4):e0175344. doi: 10.1371/journal.pone.0175344 (PMC5396893; doi:10.1371/journal.pone.0175344)
Supplement: S3 Table — (PDF) [file pone.0175344.s003.pdf]

S3 Table. Chemicals reported to the SCAQMD and used in routine oil and gas production for which experimental toxicity information could not be located (N=97).

Chemical name	CASRN
MBNPA (2-bromo-3-nitrilopropionamide)	1113-55-9
1-tetradecene	1120-36-1
Silica gel	112926-00-8
Quaternary ammonium compounds, benzyl(hydrogenated tallow alkyl)dimethyl, stearates, salts with bentonite	121888-68-4
Mullite	1302-93-8
1,2-Ethanediaminium, N1,N2-bis[2-[bis(2-hydroxyethyl)methylammonio] ethyl]-N1,N2-bis(2-hydroxyethyl)-N1,N2-dimethyl-, chloride (1:4)	138879-94-4
Cristobalite	14464-46-1
Talc	14807-96-6
Vinylidene chloride/methylacrylate copolymer	25038-72-6
Polyacrylamide	26006-22-4
Monoethanolamine borate (1:x)	26038-87-9
1-eicosene	3452-07-1
Polyepichlorohydrin, trimethyl amine quaternized	51838-31-4
1,2,3-Trimethylbenzene	526-73-8
Silanetriol, 1-(3-aminopropyl)-	58160-99-9
Ethyloctynol	5877-42-9
Silicon dioxide crystalline	60676-86-0
Formaldehyde, polymer with 2-methyloxirane, 4-nonylphenol and oxirane	63428-92-2
Glassy calcium magnesium phosphate	65997-17-3
Alcohols, C10-14, ethoxylated	66455-15-0
Silanetriol, (3-aminopropyl)-, homopolymer	68400-07-7
Thiourea, polymer with formaldehyde and 1-phenylethanone	68527-49-1
Benzenesulfonic acid, c10-16-alkyl derivs., compds. with 2-propanamine	68584-24-7
Benzenesulfonic acid, c10-16-alkyl derivs., compds. with triethanolamine	68584-25-8
Hydrocarbons, terpene processing by-products	68956-56-9
Petroleum distillates	68990-35-2
Tricalcium phosphate	7758-87-4
Lecithins	8002-43-5
Sulfuric acid, calcium salt, hydrate (2:2:1)	10034-76-1
1-propanesulfonic acid, 2-methyl-2-[(1-oxo-2-propenyl)amino]-, monoammonium salt, polymer with 2-propenamide	110897-64-8
Mica	12001-26-2
Gilsonite	12002-43-6
Canola oil	120962-03-0
Potassium oxide	12136-45-7
Aluminum oxide silicate	12141-46-7
Sodium oxide	12401-86-4
Diutan	125005-87-0
Undecanol, branched and linear	128973-77-3
Sodium aluminate	1302-42-7
Calcium oxide	1305-78-8
Limestone	1317-65-3
Saponite	1319-41-1
Dodecyl(2-hydroxy-3-sulfonatopropyl)dimethylammonium	13197-76-7
Pumice	1332-09-8
Gypsum	13397-24-5
Lithium chlorate	13453-71-9
Humic acid	1415-93-6

Chemical name	CASRN
1-propanaminium, n-(carboxymethyl)-n,n-dimethyl-3-(((13z)-1-oxo-13-docosenyl)amino)-, inner salt	149879-98-1
Trydymite	15468-32-3
Cyclohexamine sulfate	19834-02-7
Acrylic polymer	203008-81-5
Acetic acid ethenyl ester, polymer with choroethene and ethene	25085-46-5
2-propenoic acid, 2-methyl-, polymer with 2-propenoic acid	25751-21-7
Plaster of paris	26499-65-0
Diisopropylnaphthalenesulfonic acid	28757-00-8
Formaldehyde, polymer with 4-(1,1-dimethylethyl)phenol, 2-methyloxirane and oxirane	30704-64-4
Calcium magnesium oxide	37247-91-9
2-butenedioic acid (e)-, polymer with 1,2-ethanediol and .alpha.,.alpha.?-[(1-methylethylidene)di-4,1-phenylene]bis[.omega.-hydroxypoly[oxy(methyl-1,2-ethanediyl)]]	39382-21-3
Coal, ground	50815-10-6
Sodium borosilicate	50815-87-7
Sodium gluconate	527-07-1
1-octanesulfonic acid sodium salt	5324-84-5
Poly(oxy-1,2-ethanediyl), .alpha.-[bis(1-methylpropyl)phenyl]-.omega.-hydroxy-	53964-94-6
Silica	61790-53-2
Petroleum resins	64742-16-1
Petroleum distillates	64742-46-7
Sulfurous acid, sodium salt (1:1), polymer with formaldehyde and 1,3,5-triazine-2,4,6-triamine	64787-97-9
Esters of rosin oligomers with pentaerythritol	65997-12-8
Portland cement	65997-15-1
Fatty acids, tall-oil, reaction products with triethanolamine	67784-78-5
4,7-methano-1h-indene, 3a,4,7,7a-tetrahydro-, polymer with 1,3-cyclopentadiene, cyclopentene, 1-hexene, 2-methyl-2-butene and 1,3-pentadiene	68003-51-0
Poly(oxy-1,2-ethanediyl), .alpha.-sulfo-.omega.-hydroxy-, c6-10-alkyl ethers, ammonium salts	68037-05-8
Poly(oxy-1,2-ethanediyl), .alpha.-isodecyl-.omega.-hydroxy-, phosphate, potassium salt	68071-17-0
Ashes (residues), coal	68131-74-8
Lignosulfonic acid, ethoxylated, sodium salts	68611-14-3
Amide surfactant	68647-77-8
Ethanol,2,2’-oxybis-,reactionproductswithammonia,morpholinederivs.residues	68909-77-3
Fumes, silica	69012-64-2
Welan gum	72121-88-1
Magnesium	7439-95-4
Tall oil	8002-26-4
Compound synthetic diesters	8029-39-8
Fuller's earth	8031-18-3
2-propenoic acid, polymer with 2-propenamide	9003-06-9
Polypropylene	9003-07-0
Cellulose, 2-hydroxyethyl ether	9004-62-0
Starch	9005-25-8
Lignin	9005-53-2
Cellophane	9005-81-6
Naphthalenesulfonate-formaldehyde condensate, sodium salt	9008-63-3
2-propenoic acid, polymer with sodium 2-propenoate	9033-79-8

Chemical name	CASRN
Maltodextrin	9050-36-6
Aluminium distearate	300-92-5
Acetic acid ethenyl ester, polymer with ethanol	25213-24-5
Benzene, tetrapropylene-	25265-78-5
L-Glutamic acid, N,N-Diacetic acid, Tetrasodium salt	51981-21-6
L-Glutamic acid, N,N-Diacetic acid	58976-65-1
