# Supplementary material for: Comparison of chemical-use between hydraulic fracturing, acidizing, and routine oil and gas development
Source: PLoS One. 2017 Apr 19;12(4):e0175344. doi: 10.1371/journal.pone.0175344 (PMC5396893; doi:10.1371/journal.pone.0175344)
Supplement: S4 Table — (PDF) [file pone.0175344.s004.pdf]

S4 Table. Chemicals reported to the SCAQMD and used in routine oil and gas development activities considered chemicals of concern based on six reference lists consulted.

Chemical name	CASRN	European Chemicals Agency Candidate List of Substances of Very High Concern	U.S. EPA Contaminant Candidate List (CCL) 4	U.S. EPA National Primary Drinking Water Standard and Health Advisory Chemicals List	Chemicals known to the state of California to cause cancer or reproductive toxicity (Prop 65)	California EPA Toxic Air Contaminant (TAC) List[a]	OSPAR Substances of Possible Concern
1,2,4-Trimethylbenzene	95-63-6			X		4b	
1,3,5-Trimethylbenzene	108-67-8			X			
1-butanol	71-36-3		X			4b	
2-Butoxyethanol (Ethylene glycol butyl ether)	111-76-2					2a	
Acetophenone	98-86-2					4a	
Acrylamide	79-06-1	X		X	X		
Aluminum	7429-90-5					4b	
Ammonium sulfate	7783-20-2					4b	
Boric acid	10043-35-3	X					
Boron sodium oxide	1330-43-4	X					
Diethanolamine	111-42-2				X	2a	
Ethylbenzene	100-41-4			X	X	2a	
Ethylene glycol	107-21-1		X	X	X	2a	
Ethylene oxide	75-21-8		X		X		
Formaldehyde	50-00-0		X	X	X	2a	
Glutaraldehyde	111-30-8					2b	
Isopropanol	67-63-0					2b	
Isopropylbenzene	98-82-8			X	X	4a	
Lithium carbonate	554-13-2				X		
Methanol	67-56-1		X		X	2a	
Naphthalene	91-20-3			X	X		
Nitrilotriacetic acid	139-13-9				X	4b	
Phosphoric acid	7664-38-2					2b	
Quinoline	91-22-5		X			5	
Silica	7631-86-9					3	
Sodium hydroxide	1310-73-2					2b	
Sulfuric acid	7664-93-9					2b	

Chemical name	CASRN	European Chemicals Agency Candidate List of Substances of Very High Concern	U.S. EPA Contaminant Candidate List (CCL) 4	U.S. EPA National Primary Drinking Water Standard and Health Advisory Chemicals List	Chemicals known to the state of California to cause cancer or reproductive toxicity (Prop 65)	California EPA Toxic Air Contaminant (TAC) List[a]	OSPAR Substances of Possible Concern
Toluene	108-88-3			X	X	2a	
Xylenes	1330-20-7			X		2a	
Poly(oxy-1,2-ethandiyl), a-(nonylphenyl)-w-hydroxy-	9016-45-9						X
Bis(isopropyl)naphthalene	38640-62-9						X

[a]**Category 2a** – Substances identified as Toxic Air Contaminants, known to be emitted in California, with one or more health values under development by the Office of Environmental Health Hazard Assessment for review by the Scientific Review Panel, **Category 2b** – Substances NOT identified as Toxic Air Contaminants, known to be emitted in California, with one or more health values under development by the Office of Environmental Health Hazard Assessment for review by the Scientific Review Panel, **Category 3** – Substances known to be emitted in California and are NOMINATED for development of health values or additional health values, **Category 4a** – Substance identified as Toxic Air Contaminants, known to be emitted in California and are TO BE EVALUATED for entry into Category III, **Category 4b** – Substance NOT identified as Toxic Air Contaminants, known to be emitted in California and are TO BE EVALUATED for entry into Category III, **Category 5** – Substance identified as Toxic Air Contaminants, and NOT KNOWN TO BE EMITTED from stationary source facilities in California
